# Synergistic antibacterial mechanism of silver-copper bimetallic nanoparticles

**DOI:** 10.3389/fbioe.2023.1337543

**Published:** 2024-01-08

**Authors:** Zhaonan Hao, Mingbo Wang, Lin Cheng, Minmin Si, Zezhou Feng, Zhiyuan Feng

**Affiliations:** ^1^ School and Hospital of Stomatology, Shanxi Province Key Laboratory of Oral Diseases Prevention and New Materials, Shanxi Medical University, Taiyuan, China; ^2^ Guangdong Engineering Technology Research Center of Implantable Medical Polymer, Shenzhen Lando Biomaterials Co, Ltd., Shenzhen, China; ^3^ Shanxi Bethune Hospital, Shanxi Academy of Medical Sciences, Tongji Shanxi Hospital, Third Hospital of Shanxi Medical University, Taiyuan, China; ^4^ Shanxi Academy of Advanced Research and Innovation (SAARI), Taiyuan, China; ^5^ Department of Orthodontics, Shanxi Provincial People’s Hospital, The Fifth Clinical Medical College of Shanxi Medical University, Taiyuan, China

**Keywords:** Ag-Cu nanoparticles, antibiotic resistance, antibacterial mechanism, nanotoxicity and genotoxicity, nanoparticle synthesis

## Abstract

The excessive use of antibiotics in clinical settings has resulted in the rapid expansion, evolution, and development of bacterial and microorganism resistance. It causes a significant challenge to the medical community. Therefore, it is important to develop new antibacterial materials that could replace traditional antibiotics. With the advancements in nanotechnology, it has become evident that metallic and metal oxide nanoparticles (MeO NPs) exhibit stronger antibacterial properties than their bulk and micron-sized counterparts. The antibacterial properties of silver nanoparticles (Ag NPs) and copper nanoparticles (Cu NPs) have been extensively studied, including the release of metal ions, oxidative stress responses, damages to cell integrity, and immunostimulatory effects. However, it is crucial to consider the potential cytotoxicity and genotoxicity of Ag NPs and Cu NPs. Numerous experimental studies have demonstrated that bimetallic nanoparticles (BNPs) composed of Ag NPs and Cu NPs exhibit strong antibacterial effects while maintaining low cytotoxicity. Bimetallic nanoparticles offer an effective means to mitigate the genotoxicity associated with individual nanoparticles while considerably enhancing their antibacterial efficacy. In this paper, we presented on various synthesis methods for Ag-Cu NPs, emphasizing their synergistic effects, processes of reactive oxygen species (ROS) generation, photocatalytic properties, antibacterial mechanisms, and the factors influencing their performance. These materials have the potential to enhance efficacy, reduce toxicity, and find broader applications in combating antibiotic resistance while promoting public health.

## Introduction

The increasing bacterial resistance due to widespread antibiotic use is alarming. Currently, global epidemics caused by various microorganisms pose a serious threat to national economic development and public safety (Ventola, 2015; Kurt Yilmaz and Schiffer, 2021). According to relevant data, bacterial resistance is a major obstacle in the fight against pathogenic microorganisms. In 2019, 1.27 million people died directly from antibiotic-resistant infections, while 4.95 million deaths were indirectly related to this issue, surpassing the number of deaths caused by AIDS or malaria (Yang et al., 2022). Therefore, the development of novel and efficient antibacterial materials is of utmost importance.

Nanoparticles effectively combat bacterial resistance via direct killing, membrane inhibition, and targeted mechanisms (Pelgrift and Friedman, 2013). Inorganic metal nanoparticles offer simplicity, lower toxicity, and reduced resistance compared to organics. Their small size, charge, and surface area enhance microorganism control (Yang et al., 2022). Developing nanoparticle resistance requires multiple genetic mutations, highlighting their potential against multi-drug-resistant microorganisms (Mohammadi et al., 2018; Arora et al., 2020). Silver nanoparticles (Ag NPs) possess remarkable antibacterial efficacy and minimal resistance propensity. They disrupt bacterial membranes, interfere with DNA and protein processes by releasing Ag^**+**^ ions, and capitalize on reactive oxygen species for enhanced microbial control (Egger et al., 2009; Piao et al., 2011; Bondarenko et al., 2013; Qing et al., 2018; Ghosh M. et al., 2021; Targhi et al., 2021; Almutairi et al., 2022). Bondarenko’s study has confirmed the ability of Ag NPs to selectively impact bacterial inner membranes, contributing to a more comprehensive understanding of their mode of action (Bondarenko et al., 2018). Numerous studies have highlighted that the antibacterial mechanism of Cu NPs primarily involves the induction of oxidative stress through the generation of reactive oxygen species (ROS), the release of metal ions, and the internalization of nanoparticles (Vincent et al., 2016; Siddiqi and Husen, 2020; Bhattacharjee et al., 2022). Usman proposed that while the effectiveness of Cu NPs is comparable to that of Ag NPs, the challenge of oxidation during their production and storage processes still persists (Usman et al., 2013). Torres observed that Ag-Cu NPs exhibit a strong synergistic antibacterial effect due to increased cell permeability. Therefore, he suggests that employing silver-copper alloy nanoparticles appears to be a cost-effective alternative to traditional antibiotics (Torres-Urquidy and Bright, 2012; Garza-Cervantes et al., 2017).

**FIGURE 1:**
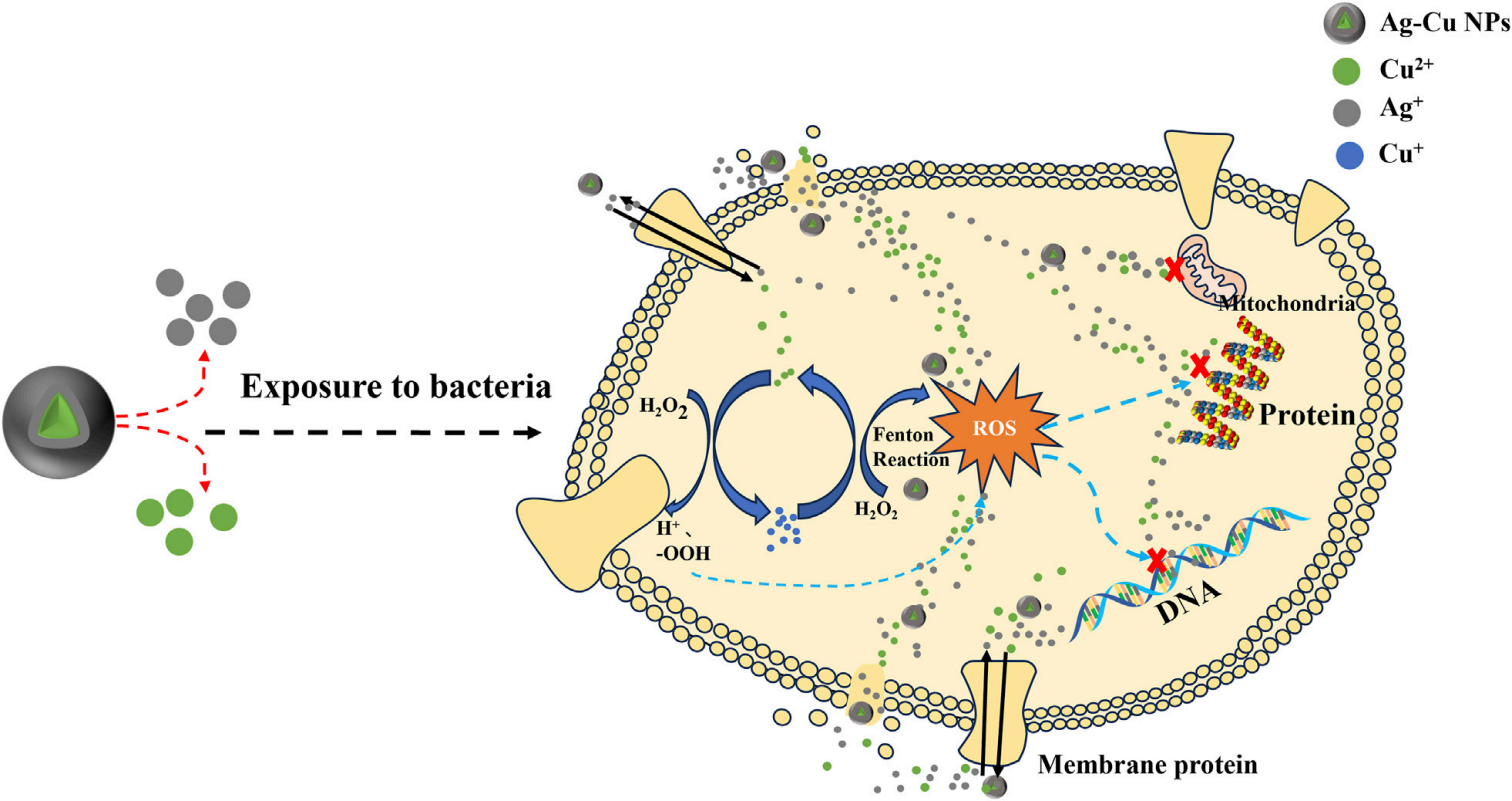
Synergistic bactericidal effect of Ag-Cu NPs.

Bimetallic nanoparticles (BNPs) have garnered considerable attention due to their exceptional physical properties, which enable the integration of different metals to enhance optical, catalytic, and antibacterial performances (Alavi and Karimi, 2018a; Malik et al., 2023). Through their experimental observations of synthesized Ag-Cu NPs, Długosz et al. discovered that these BNPs exhibited minimal genotoxicity and sustained antibacterial efficacy. Consequently, they proposed the incorporation of silver and copper nanoparticles into the BNPs system as a promising avenue for achieving more effective and safer antimicrobial applications (Długosz et al., 2021). Numerous researchers have demonstrated that Ag-Cu NPs address the limitations of individual copper and silver nanoparticles, amplifying antibacterial effects, improving stability, and mitigating nanotoxicity concerns (Perdikaki et al., 2016; Kalińska et al., 2019; Sabira et al., 2020). These findings hold significant implications for advancing antimicrobial products and guiding future research endeavors in this field. The elucidation of the precise bactericidal mechanism of Ag-Cu NPs is presented in Figure 1.

This review highlights the unique antibacterial synergy between silver nanoparticles and copper nanoparticles. The paper explores the effects of various synthesis methods, structures, carriers, pH, and photocatalytic mechanisms on the antibacterial performance of Ag-Cu NPs. The aim of this paper is to provide a comprehensive theoretical foundation for future studies in this field.

## Synthesis method of Ag-cunps

Extensive research indicates that the distribution of metals in BNPs is significantly influenced by the preparation process, leading to the formation of diverse structures. The general strategies for synthesizing nanomaterials typically involve either a “top-down approach” or a “bottom-up approach.” (Sharma et al., 2019).

The top-down approach involves the use of physical techniques (e.g., pulsed laser ablation in liquids, grinding) or chemical methods (e.g., chemical alloying) to break down larger targets (e.g., rods, sheets or films) into smaller ones. In this process, the addition of stabilizers is selectively employed to prevent aggregation, resulting in the production of nanomaterials (Nyabadza et al., 2023). However, research indicates shortcomings in the precise control of BNP size and shape using these methods. The fragmentation of bulk materials often leads to non-uniform surface topography or edge fractures, thereby affecting the physical and chemical properties of BNS (Bhol et al., 2020).

**FIGURE 2:**
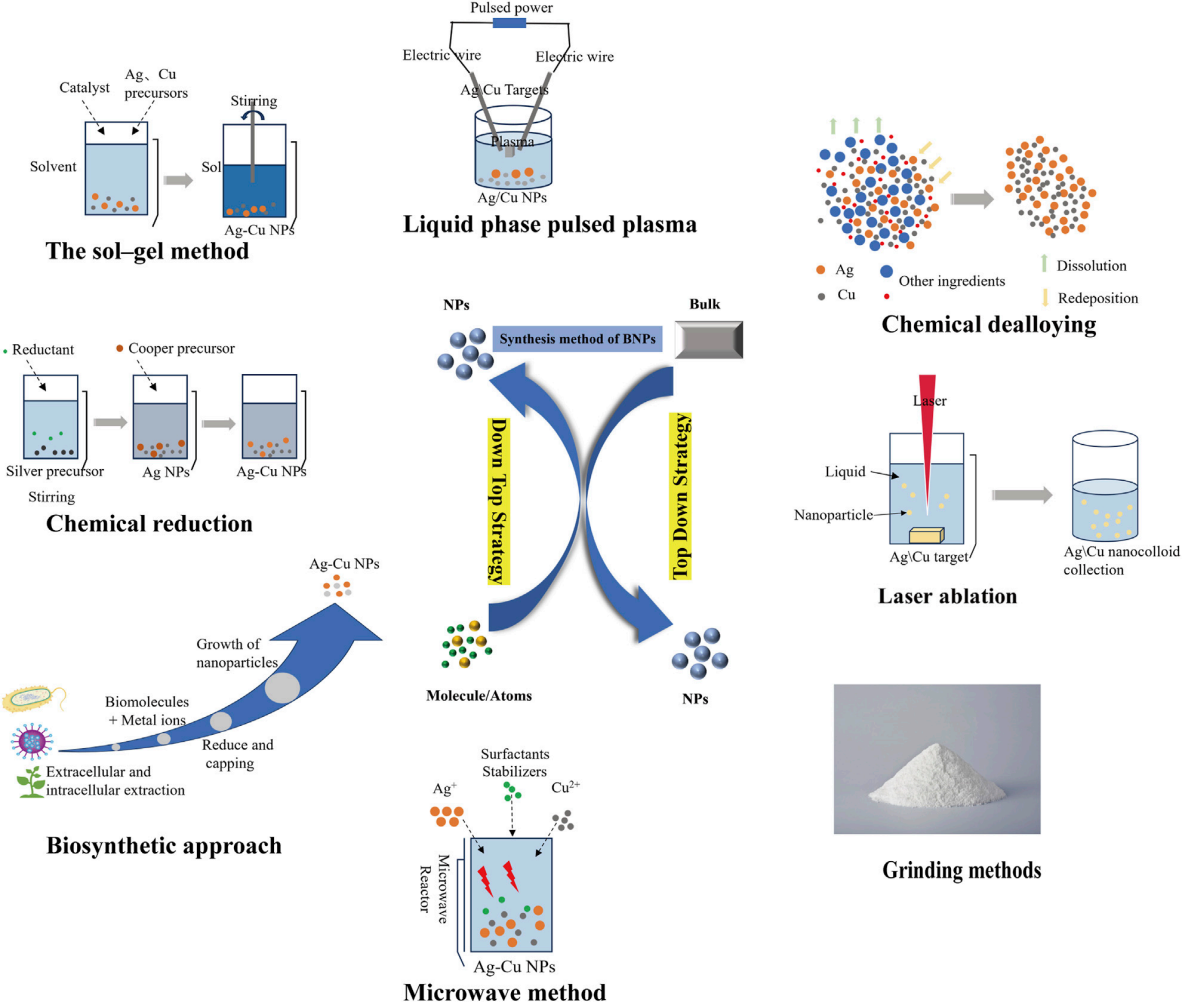
Synthesis methods of BNPs.

In contrast, the bottom-up approach involves assembling atoms into nanoparticles (NPs) through physical methods (e.g., aerosol processing), chemical methods (e.g., chemical reduction, displacement reactions), or bio-synthesis methods (Nyabadza et al., 2023). In this approach, nanostructures are assembled from the bottom up through stacking interactions between atoms and molecules, forming uniformly distributed structural units. This method initially reduces precursors to atoms, followed by nucleation and growth processes. Researchers can precisely control the size and shape of the desired product by adjusting composition parameters, offering advantages such as absolute precision, complete process control, and minimal energy loss (Ghosh Chaudhuri and Paria, 2012; Bhol et al., 2020). While it is noteworthy that the bottom-up approach generally exhibits a slower generation rate, it has been convincingly demonstrated to be more advantageous for nanoparticle synthesis when compared to the top-down approach (Ghosh Chaudhuri and Paria, 2012). Traditionally, silver-copper nanoparticles have been predominantly synthesized using chemical or physical methods. Although there are methods involving biological synthesis, they have not been widely adopted, potentially indicating a future trend. Table 1 summarizes common synthesis methods for Ag-Cu NPs, and Figure 2 illustrates several common synthesis approaches of BNPs. Next, we will primarily discuss several common methods for synthesizing Ag-Cu NPs from the perspective.

**TABLE 1:** Summarized different synthesis routes for Ag-Cu NPs.

Synthesis method	Precursors	Research objective	Nano structure	Pros/Cons	References
Reduction reaction (Down-Top)	AgNO_3_、Cu(NO_3_)_2_·3H_2_O	Antibiotic resistance	Alloy NPs	Simple process, catalytic activity, low cost, optimal control of BNP size and distribution *via* experiment parameter tuning**/**Toxic precursors, environmentally highly toxic, elevated reaction conditions	Pourjafari et al. (2022)
	AgNO_3_、Cu(NO_3_)_2_·XH_2_O	Bactericidal agent	Alloy NPs		Zain et al. (2014)
	Ag NPs、Copper Nitrate	Antibiotic resistance	Alloy NPs		Zhou et al. (2022)
	AgNO_3_、Cu(I)Cl	Bactericidal agent	Alloy NPs		Jang et al. (2020)
	AgNO_3_、 Cu(NO_3_)_2_·3H_2_O	Bactericidal agent	Core-Shell NPs		Sabira et al. (2020)
	AgNO_3_、CuCO_3_	Antibiotic resistance	Core-Shell NPs		Valdez-Salas et al. (2021)
	AgNO_3_、CuSO_4_	Antibiotic resistance	Alloy NPs		Mohammadi et al. (2018)
	AgNO_3_、Cu(NO_3_)_2_·3H_2_O	Bactericidal agent	Alloy NPs		Valodkar et al. (2011)
Dealloying approach (Top-Down)	Zr_48_Cu_36_Ag_8_Al_8_ MG ribbon	Better performance expression	Core-Shell NPs	Simple, cost-effective, efficient, product stability and purity, no surfactants**/**Reaction conditions sensitivity	Liu et al. (2017)
	Mg_65_Ag_12.5_Cu_12.5_Y_10_ metallic glass	Better performance expression	Alloy NPs		Li et al. (2017)
	Mg–(Ag,Cu)–Y metallic glasses	Bactericidal agent	Alloy NPs		Wang et al. (2019)
Pulsed Laser Ablation (Top-Down)	Ag/Cu alloy targets	Optical and photoelectric properties	Alloy NPs	Highly reactive surface, pure products, precise control of particle size, yield, and shape**/**strict laser parameter control	Satya Bharati et al. (2019)
	Ag/Cu alloy targets	Study of Structural Transformations	Alloy NPs		Malviya and Chattopadhyay (2014)
	Ag/Cu alloy targets	Study of Synthesis Methods	Alloy NPs		Ahmadinejad and Mahdieh (2022)
The vapor deposition technique (Down-Top)	AgNO_3_、CuCl_2_	Antibiotic resistance	Alloy NPs	Uniform size, controllable composition, precise control of center-to-center spacing	Perdikaki et al. (2016)
	**————————**	Better performance expression	Alloy NPs		Bogatyrenko et al. (2023)
	Ag, Cu, and Mg Rods	Bactericidal agent	Cluster-in-Cluster Form		Benetti et al. (2019)
Microwave reactor (Down-Top)	AgNO_3_、CuSO_4_·5H_2_O	Bactericidal agent	Alloy NPs	Short reaction times, small particle sizes, high purity and uniformity**/**Longer initial sample preparation	Długosz et al. (2021)
	AgNO_3_ and CuNO_3_	Bactericidal agent	**—————**		Ameen (2022)
	AgNO_3_、Cu(CO_2_CH_3_)_2_·H2O	Study of optical properties	Core-Shell NPs		Xiong et al. (2016)
Biosynthetic approach (Down-Top)	AgNO_3_、Cu(CH_3_COO)_2_	Bactericidal agent	Alloy NPs	Rapid reaction, cost-effective, environmentally friendly, excellent candidates for biological applications**/**Electronic applications impeded	Kumar et al. (2023)
	AgNO_3_、CuSO_4_	Bactericidal agent	Core-Shell NPs		Hamouda et al. (2023)
	AgNO_3_、Cu(NO_3_)_2_·3H_2_O	Antibiotic resistance	Alloy NPs		Malik et al. (2023)
	AgNO_3_、CuSO_4_	Antibiotic resistance	Alloy NPs		Alavi and Karimi (2018a)
	AgNO_3_、CuSO_4_	Dye pollution prevention	Alloy NPs		Kushwah et al. (2019)
	AgNO_3_、Cu(OAc)_2_·H_2_O	Better performance expression	Alloy NPs/Core-Shell NPs		Tsuji et al. (2010)
The sol–gel method (Down-Top)	AgNO_3_、Cu(NO_3_)_2_·5H_2_O、Zn(CH_3_COO)_2_	Performance Investigation	**—————**	High repeatability and efficiency 、aerogel synthesis; long reaction times, intricate steps, high skill demands, limited control over NP size	Modwi et al. (2021)
	Ag、Cu、Au Targets	Performance Investigation	Alloy NPs		Zhang et al. (2018a)
	AgNO_3_、Cu(NO_3_)_2_·3H_2_O Zn(NO_3_)_2_	Performance Investigation	Alloy NPs		Ziabka et al. (2023)

### The top down approach

#### Chemical dealloying

Chemical dealloying is one of the most commonly used and traditional techniques for synthesizing anisotropic bimetallic nanostructures. This method is characterized by its simplicity, cost-effectiveness, and the absence of surfactants or other adsorbates. By selectively etching one or more noble metal elements from an alloy, this approach results in the formation of binary nano-composites with exceptionally clean surfaces (Qiu et al., 2011). However, there are limited reports on the preparation of BNPS using the chemical dealloying method, particularly Ag-Cu NPs. Through chemical dealloying of Zr-Cu-Ag-Al-O amorphous/crystalline composite material, Liu et al. successfully synthesized a novel Cu-supported Ag-Cu nano-porous alloy. This alloy not only exhibits outstanding air stability at room temperature but also demonstrates significantly improved oxidation stability compared to previously reported Cu-Ag core-shell microparticles (Liu et al., 2017). On another front, Li et al. notably achieved efficient preparation of a chemically homogeneous three-dimensional nano-porous bimetallic Ag-Cu alloy by subjecting metallic glass Mg_65_Ag_12.5_Cu_12.5_Y_10_ to dealloying treatment in dilute H_2_SO_4_ aqueous solution under self-corrosion conditions (Li et al., 2017).

#### Pulsed laser ablation

As a top-down method for nanoparticle fabrication, Pulsed Laser Ablation in Liquids (PLAL) involves irradiating solid targets (typically rods, sheets, or thin films) in a liquid medium. This method allows the liquid to collect the ejected nanoparticles produced by laser processing, forming a colloidal suspension applicable across various fields. PLAL, as an exemplary approach for catalytic synthesis of nanoparticles, yields clean, uncapped, and surfactant-free nanoparticles with highly reactive surfaces. Compared to several prevalent manufacturing techniques, PLAL offers faster production of BNPs and effective control over the size distribution, yield, and shape of nanoparticles (Fazio et al., 2020; Nyabadza et al., 2023). However, its drawbacks primarily revolve around the necessity to determine suitable laser processing parameters, demanding extensive experimental work, and the existence of intricate, nonlinear relationships between input and output factors (e.g., NP size) (Nyabadza et al., 2023).

Recently, Bharati et al. synthesized silver-copper alloy nanoparticles (NPs) using the Pulsed Laser Ablation in Liquids method. Their research underscores the potential of Ag-Cu alloy NPs in detecting various analyte molecules (Satya Bharati et al., 2019). In a previous study, Malviya et al. observed a transition in the morphology of Ag-Cu alloy synthesized by the PLAL method, from a biphasic structure to a dispersed morphology within the nanoparticles, and ultimately to a core-shell structure, with increasing copper concentration. This study provides valuable insights into the application of the PLAL synthesis method for Ag-Cu NPs (Malviya and Chattopadhyay, 2014).

### The down top approach

#### Chemical reduction method

Chemical reduction, as one of the most common bottom-up methods for synthesizing BNPs, enables precise control over BNPs’ size and distribution through meticulous control of experimental parameters. It offers simplicity, scalability, and the seamless integration of foreign atoms into the synthesis process (Paszkiewicz et al., 2016; Dlamini et al., 2023). However, significant challenges remain. Chemical reduction often requires the separation of unreacted reagents and impurities from the resulting NPs, and the use of potentially harmful precursors is commonplace. Additionally, reaction times can be prolonged, and some conditions may be stringent, including very high reaction temperatures, posing challenges for practical implementation (Nikam et al., 2018).

Valdez-Salas et al. suggest that the advantages of this method for the synthesis of Ag-Cu NPs are mainly due to the fact that it can strategically manipulate the arrangement of arrays of different core-shell structures and take advantage of the directionality provided by core-shell elements, the sequence of chemical reduction reactions influences the structure of the synthesized Ag-Cu NPs (Valdez-Salas et al., 2021). Manikam et al. suggested that nanoparticle synthesis via chemical reduction can be divided into two subgroups: the first involves co-reduction of two different metal salts, while the second involves successive reduction of two metal salts (Manikam et al., 2011). The consecutive reduction of metal salts readily produces core-shell nanostructures, while simultaneous reduction of metal salts increases the likelihood of forming nanoalloys or nanoclusters (Mvango and Mashazi, 2019).

#### Liquid phase pulsed plasma method

The liquid-phase pulsed plasma method has been investigated as an economical, environmentally friendly approach for synthesizing nanoparticles. In the synthesis of BNPs, this method addresses challenges associated with traditional chemical reduction methods, particularly in handling large phase-segregated mixtures. Liquid-phase pulsed plasma has demonstrated efficacy in producing non-equilibrium nanoscale particles with exceptionally high cooling rates (Yang L. et al., 2019). This method not only enables the generation of nano-materials with varied sizes and controlled shapes but also offers a streamlined process with low energy consumption. Importantly, it often eliminates the use of toxic precursors or reagents (Ma et al., 2020). Limited research exists on the synthesis of Ag-Cu NPs using liquid-phase pulsed plasma. In a recent study, Yang et al. successfully employed this method to achieve the uniform alloying of Ag-Cu NPs. Application of these nanoparticles in antibacterial research revealed their remarkable ability to completely suppress the growth of *Escherichia coli* and *Staphylococcus aureus* at ultra-low concentrations within a brief incubation period. Importantly, the nanoparticles exhibited minimal genetic toxicity (Yang L. et al., 2019). This study presents a promising avenue for the preparation of silver-copper nano-composite materials.

#### Microwave method

Microwave (MW) method is a bottom-up physical approach. This method involves mixing precursors and pH stabilizers (e.g., through magnetic stirring), and then subjecting the mixture to microwave irradiation for a specific duration (usually a few minutes) to form nanoparticles. The size and yield of the nanoparticles depend on the time and power of microwave radiation. While this process requires low-cost equipment, it often entails a relatively lengthy sample preparation time (Li et al., 2014).

Microwave method has piqued interest in colloidal NP synthesis due to the “specific MW effect.” his unique effect encompasses uniform heating and rapid reaction rates, not achievable through traditional methods. They play a crucial role in controlling the balance of nucleation and growth processes in liquid media, essential for forming NPs with predetermined structural, compositional, and geometric features (Chen et al., 2013). Xiong et al. compared the nanostructure and optical properties of Ag-Cu NPs synthesized using microwave and traditional oil bath heating methods. They observed that Ag-Cu NPs produced via microwave reaction exhibit smaller particle sizes, higher purity, and greater uniformity. Additionally, the microwave synthesis method minimizes the introduction of toxic chemicals, rendering it a more environmentally friendly option (Xiong et al., 2016).

#### The sol–gel method

The sol-gel method, a bottom-up approach, entails blending precursors with a solvent in the presence of a catalyst to create a homogeneous solution. The addition of water initiates hydrolysis, leading to the formation of suspended particles known as sol. Noteworthy advantages of this method include the convenient synthesis of aerogels and the ability for large-scale nanoparticle production. However, it demands skilled chemists, involves multiple processing steps, and provides limited control over nanoparticle size (Nyabadza et al., 2023).

Research on the sol-gel synthesis of Ag-Cu NPs is extensive, with recent studies often combining Ag-Cu NPs with other metallic elements. The focus lies primarily on coating preparation and the investigation of optical and electrical properties. For instance, Modwi and colleagues found that Ag-modified Cu-doped ZnO nanoparticles exhibit superior electrical characteristics (Modwi et al., 2021). Meanwhile, Zhang et al. discovered that under near-infrared light, NYFT composite materials loaded with Au-Ag-Cu demonstrate heightened photocatalytic activity and increased effectiveness against *S. aureus* (Zhang H. et al., 2018).

### Biosynthetic approach

Although physical and chemical methods have successfully produced high-purity nanoparticles of desired sizes, these processes often incur high costs and involve toxic chemicals. Hence, the toxicity issues in the preparation process become particularly critical. One of the primary goals of nanotechnology is to establish an ecologically friendly production process. To achieve this, some researchers have focused on biological methods for synthesizing metal nanoparticles due to their advantages, such as rapidity, cost-effectiveness, and environmental friendliness (Ghosh S. et al., 2021).

Nanoparticles (NPs) biosynthesis is fundamentally a bottom-up process involving the reduction of ions in aqueous solutions. In these studies, proteins and enzymes secreted by organisms like plants, bacteria, fungi, and yeast play a crucial role as reducing agents in NP synthesis. Although NPs synthesized through biological routes are enveloped by proteins and enzymes, making them excellent candidates for biological applications like drug delivery, this encapsulation may hinder their application in the field of electronics (Nyabadza et al., 2023).

However, research on the green synthesis of Ag-Cu NPs is relatively scarce. Recently, Hamouda et al. conducted a study evaluating the antibacterial activity of Ag-Cu NPs extracted from the seaweed *Ulva lactuca* against both Gram-positive and Gram-negative bacteria, as well as their impact on antibiofilm formation. The synthesized nanoparticles exhibited significant antibacterial activity against multidrug-resistant strains, highlighting their potential as alternative antibacterial agents (Hamouda et al., 2023). Simultaneously, Kumar et al. successfully prepared Ag-Cu NPs from wastewater extract of *Prosopis cineraria pods* (commonly known as “Sangri”), confirming the promising application prospects of this synthesis method (Kumar et al., 2023). Other plant extraction methods, such as *Artemisia haussknechtii leaf extract*, (Alavi and Karimi, 2018b), *Salvia officinalis*, (Malik et al., 2023), and *Aegle marmelos and Citrus limetta fruit peel extract* (Kushwah et al., 2019) have also been reported. The majority of these studies have focused on research in the field of antibacterial properties.

In addition to the common methods for synthesizing Ag-Cu NPs mentioned above, there is widespread interest in various bottom-up synthesis approaches, such as galvanic deposition, (Mahara et al., 2016), galvanic displacement, (Muzikansky et al., 2013; Lee et al., 2015), and one-pot synthesis (Giorgetti et al., 2013; Delsante et al., 2015). In contrast, top-down synthesis methods have received relatively less attention in the research.

## Different structures of Ag-cunps

**FIGURE 3:**
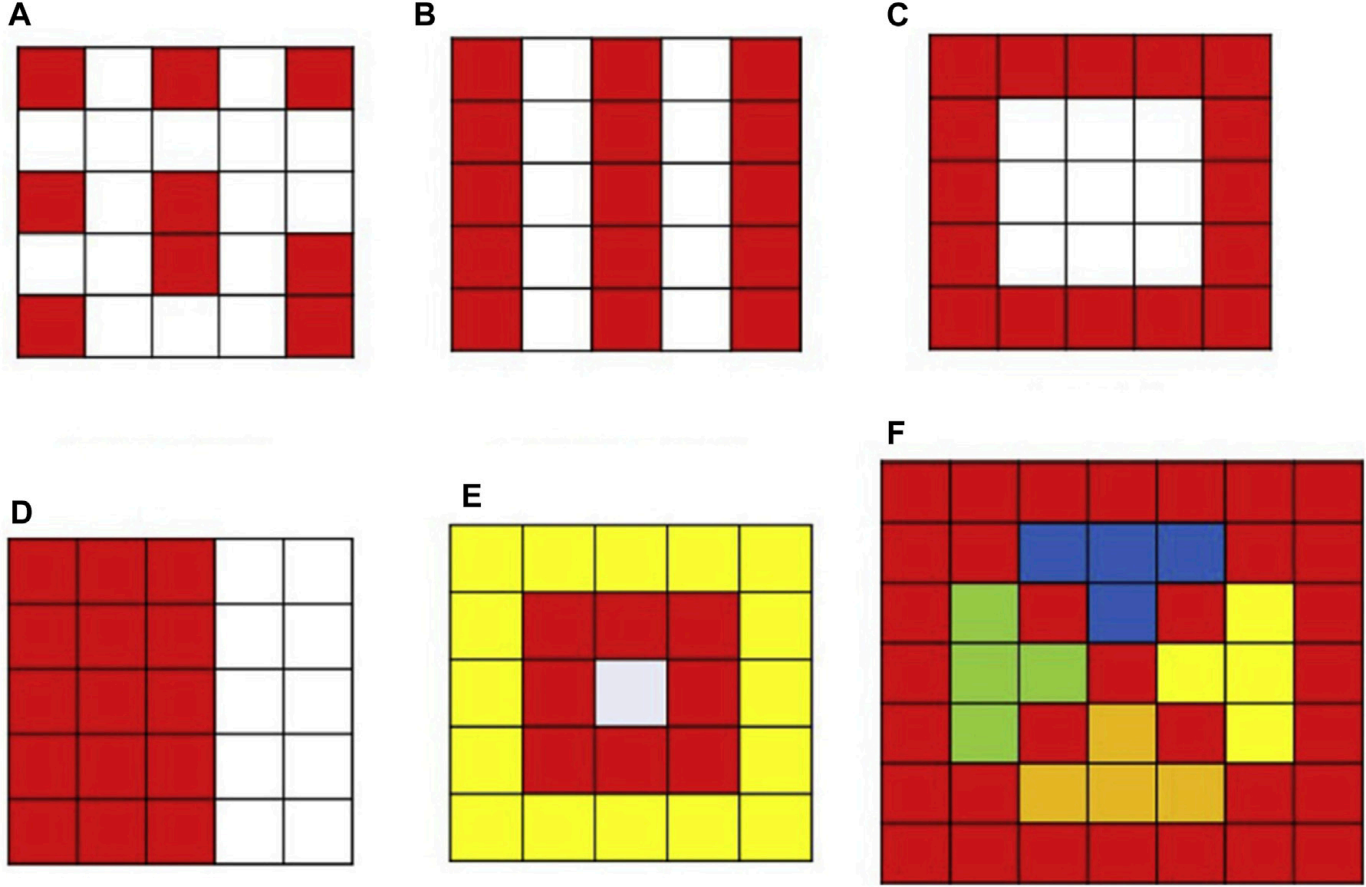
Different types of bimetallic nanostructures (NS). **(A)** Alloyed NS (random), **(B)** intermetallic NS (ordered), **(C)** core-shell NS, **(D)** subcluster NS, **(E)** multishell core-shell NS, **(F)** multiple core-single shell NS (Behera et al., 2020).

The varied morphology of nanoparticles significantly influences their antibacterial efficacy and genotoxic potential (Mohammadi et al., 2018). Research indicates that in bimetallic complexes, such as silver and copper, the interaction occurs not only at a mechanical level but also at an atomic and lattice level (Dehghani et al., 2019). Based on the arrangement of atomic structures, BNPs can be categorized into two main types: hybrid structures and segregated structures. Hybrid structures are further classified into random structures and ordered structures. Random structures are also referred to as alloy structures, while hybrid structures with ordered arrangements are known as intermetallic structures. Segregated structures consist of two different metals, with a shared interface termed as cluster structures, and a structure where one metal encases another is termed as core-shell structures (Figure 3) (Behera et al., 2020). Medynska further delineated BNPs into nine distinct nanostructure arrangements (Zaleska-Medynska et al., 2016). Recent studies have extensively explored Ag and Cu nanocomposites. Most prepared Ag-Cu nanocomposites exhibit “phase-separated” or “core-shell” configurations (Yang L. et al., 2019). The most widely reported nanocomposites have core-shell structures (Sharma et al., 2019).

### The core-shell structures

Extensively studied for their geometric control and exceptional physical properties, core-shell nanoparticles (CS NPs) exhibit synergistic attributes (Tao et al., 2017). Ferrando’s research revealed that components with higher REDOX potential in the reduction process tend to assume the core role, while the second component has a propensity to deposit onto this core, forming a core-shell structure. Furthermore, he observed that the introduction of surfactants can alter the deposition sequence, leading to the formation of an antinuclear shell arrangement (Ferrando et al., 2008). Numerous studies focus on Ag-Cu CS NPs, with concentric spherical CS NPs being most explored (Yang Z. et al., 2019). Ghosh outlines two synthesis methods for diverse CS NPs: using distinct core shapes for physical control or employing capping agents, polymers, or reagents to govern growth direction and size (Ghosh Chaudhuri and Paria, 2012). Although generally less biologically active than singular nanoparticles, CS NPs exhibit prolonged metal ion release for extended bactericidal efficacy (Feng et al., 2021). Ag-Cu CS NPs exhibit broad-spectrum antibacterial activity and negligible genetic toxicity, highlighting their potential in antimicrobial applications (Długosz et al., 2021).

Ghosh discovered that different core shapes can lead to distinct Ag-Cu NPs, and the antibacterial effectiveness and toxicity of CS NPs are intimately linked to the core shape and shell thickness (Ghosh Chaudhuri and Paria, 2012). Gan’s findings reveal that triangular CS NPs, compared to spherical and cubic counterparts, possess larger specific surface areas and porosity, thereby enhancing their antibacterial activity (Gan et al., 2018). Caruso and colleagues have observed that shell materials not only enhance core stability and dispersion through surface charge modification but also shield the core from external stimuli by adjusting shell thickness at the nanoscale (Caruso, 2001). This synergistic mechanism imparts excellent antibacterial properties to Ag-Cu NPs while effectively preventing nanoparticle aggregation and inhibiting the genetic toxicity of BNPs.

Tojo proposed that the morphology of CS NPs can be finely tuned by adjusting the proportions and introduction sequence of metal precursors (Tojo and Vila-Romeu, 2014). Notably, Osowiecki et al. discovered an intriguing phenomenon during the synthesis of Ag-Cu core-shell NPs: varying the atomic fraction of Ag leads to distinct structures, ranging from nano crescent to complete core-shell configurations with enhanced surface coverage, a novel finding (Osowiecki et al., 2018). Jang et al. demonstrated that successful formation of Ag-Cu CS NPs requires sequential reduction of Cu and Ag precursors, while simultaneous introduction or initial reduction of Ag precursors impedes the formation of Cu-Ag CS NPs. This phenomenon might be attributed to the underlying chemical reactions:

$$2Ag^{+}+Cu\to 2Ag+Cu^{2+}$$

Jang et al. (2020) Moreover, the inclusion of appropriate capping agents, surfactants, and coordination compounds has been demonstrated to have a substantial impact on the structure, shape, size, and characteristics of CS NPs (Zhao et al., 2022; Boas et al., 2023).

Several specific core-shell structures of Ag-Cu nanoparticles have been reported, including core-shell-shell particles (Bouazizi et al., 2018), metal Ag-Cu core-shell clusters with incomplete fractional phases (Benetti et al., 2019), cluster-within-cluster structures, triple onion-like core-shell structures, (Ferrando et al., 2008), multiple core-shell microspheres, and hollow core-shell structures (Ghosh Chaudhuri and Paria, 2012). These structures frequently exhibit outstanding antibacterial properties, concurrently mitigating the adverse reactions attributed to nanocomposites.

### The hybrid structures

Hybrid structures can adopt either ordered or random arrangements. When the atomic sizes of the two elements closely resemble each other, the resulting alloy tends to exhibit a random configuration. Conversely, when there is a significant disparity in the sizes of the metal atoms, and the molar ratio of the two elements is simple enough, an intermetallic compound forms, resulting in a distinct structure (Zaleska-Medynska et al., 2016).

Much like the intricate factors governing CS nanostructures, those shaping alloy nanostructures are equally diverse. An array of synthesis conditions, encompassing variations in reduction potential (Tojo and Vila-Romeu, 2014), precise control of calcination temperature (Cybula et al., 2014), modulation of radiation dose (Treguer et al., 1998), and meticulous management of precursor concentration and sequence (Tojo and Vila-Romeu, 2014), collectively contribute to the diversity observed in bimetallic alloy nanoparticle structures.

The physicochemical attributes of BNPs are governed by a diverse array of factors, spanning from precursor concentration and sequential introduction to the chosen synthesis modality, bimetallic composition, and the utilization of surfactants and terminal agents. Furthermore, extraneous parameters like variance in reduction potential, temperature, and radiation dosage throughout the synthesis procedure significantly impact the eventual nanoparticle attributes.

## Enhanced antimicrobial efficacy and reduced toxicity

### Enhanced antimicrobial properties

Bimetallic nanoparticles are highly prized for their significant potential across various fields. A multitude of studies has consistently demonstrated that BNPs inhibit bacterial growth through the following mechanisms: i) Adhesion to the cell membrane: BNPs can change the structure of the membrane, leading to altered permeability and deficiencies in cell functions such as ATP secretion and transport activity. ii) Penetration inside the cell and nucleus: BNPs can disrupt mitochondrial function, destabilize and denature proteins, destabilize ribosomes, and interact with DNA. iii) Cellular toxicity and ROS generation: BNPs have the ability to induce cellular toxicity and generate reactive oxygen species (ROS), which can oxidize proteins, lipids, and DNA bases. iv) Modulation of cellular signaling: BNPs can modify the phosphotyrosine profile, thereby influencing cellular signaling pathways (Abbasi et al., 2018; Kushwah et al., 2019; Fanoro and Oluwafemi, 2020; Medina-Cruz et al., 2020).

Van Hengel et al. discovered that in Minimum Inhibitory Concentration (MIC) and Minimum Bactericidal Concentration (MBC) experiments, the synergistic effect of Ag-Cu NPs was significantly enhanced, with a respective increase of 2 and 10 times (van Hengel et al., 2020). The underlying mechanisms involve the production of more endogenous ROS, leading to a stronger oxidative stress response and reduced bacterial cell activity (Xie et al., 2022). Furthermore, Ag-Cu NPs release higher quantities of metal ions (Zhou et al., 2022), display a more dispersed structure (Pourjafari et al., 2022), diverse morphologies and sizes (Gu et al., 2018), enhance cellular permeability in prokaryotes (Garza-Cervantes et al., 2017), and the charge transfer effect further amplifies their antibacterial activity (Yang L. et al., 2019). In the review, the minimum inhibitory concentration (MIC) and minimum bactericidal concentration (MBC) were utilized as crucial parameters for evaluating the bactericidal activity of bimetallic nanoparticles (BNPs), and Table 2 presents the antibacterial effect of Ag-Cu NPs in the retrieved articles.

**TABLE 2:** Antibacterial effect of Ag-Cu NPs in the retrieved articles.

BNPs	Bacteria	MIC (mg/L)	MBC(mg/L)	References
Ag-Cu NPs	*Bacillus subtilis*	0.054	**—**	Kalińska et al. (2019)
	*E. coli*	0.076	**—**	
	*S. aureus*	**—**	**—**	
Ag-Cu NPs	*P. aeruginosa*	1.25	2.5	Pourjafari et al. (2022)
Ag-Cu NPs	*E. coli*	0.23–0.25	0.65–0.8	Valodkar et al. (2011)
	*S. aureus*	0.3–0.34	1.2–1.6	
Ag-Cu NPs	*P. aeruginosa*	≥125	**—**	Valdez-Salas et al. (2021)
	*S. marscescens*	>500	**—**	
	*S. enterica*	>250	**—**	
	*E. coli*	>500	**—**	
	*S. aureus*	>500	**—**	
Ag-Cu NPs	*E. coli*	20	**—**	Ghosh et al. (2021a)
	*S. aureus*	20	**—**	
Ag-Cu NPs	*E. coli*	260–270	300–350	Długosz et al. (2021)
	*S. aureus*	290–350	300–400	
	*C. albicans*	350–400	450–460	
Ag-Cu NPs	*E. coli*	20	**—**	Yang et al. (2019a)
	*S. aureus*	2	**—**	
Ag-Cu NPs	*E. coli*	2.5	**—**	Wang et al. (2019)
	*S. aureus*	3	**—**	
Ag-Cu NPs	*E. coli*	15	**—**	Malik et al. (2023)
	*S. aureus*	5	**—**	
	*K.pneumoniae*	10	**—**	
	*S.epidermidis*	5	**—**	
Ag-Cu NPs	*E. coli*	0.054	**—**	Zain et al. (2014)
	*B. subtilis*	0.076	**—**	
Ag-Cu NPs	*E. coli*	500	250	Mohammadi et al. (2018)
	*L. monocytogenes*	250	250	
	*B. cepacian*	250	250	
Ag-Cu NPs	*E. coli*	0.22	0.5	Kushwah et al. (2019)
	*S. aureus*	0.3	0.9	
Ag-Cu NPs/Cu-Ag NPs	*E. coli*	25	50	Alavi and Karimi (2018a)
	*S. aureus*	5–10	10–20	
	*P. aeruginosa*	0–10	0–20	
Ag-Cu NPs/Cu-Ag NPs	*E. coli*	**—/**75	75/75	Sabira et al. (2020)
	*S. aureus*	100/75	100/75	
	*P. aeruginosa*	100/20	100/20	

### Negligible genotoxicity

The fundamental mechanisms underlying NPS (nanoparticle) toxicity encompass oxidative stress, inflammatory response, immunotoxicity, with genotoxicity being the primary factor impacting human host cells (Magdolenova et al., 2014). The mechanisms underlying nanoparticle-induced genotoxicity remain a subject of intrigue, with the degree of their impact on DNA specificity remaining unclear. Barnes et al. put forward the hypothesis that genotoxicity may arise from interactions between nanoparticles and genetic material or result from damage induced by reactive oxygen species (ROS) generated by nanoparticles or the release of toxic ions from soluble NPs (Barnes et al., 2008). Agnihotri et al. propose that nanoparticle-induced inflammation may give rise to secondary genotoxicity. In this process, activated phagocytes like neutrophils and macrophages release reactive oxygen species (ROS) that initiate oxidative damage to host cell DNA (Magdolenova et al., 2014; Agnihotri et al., 2020).

Ag NPs and Cu NPs exhibit potent antibacterial potential and have been extensively studied. The most widely accepted genotoxic mechanisms currently encompass direct DNA interactions, protein engagement, and the induction of oxidative stress, potentially resulting in host DNA damage and cellular dysfunction (Magdolenova et al., 2014). Ag NPs might enter mammalian cells via Ag^**+**^ release, generating reactive oxygen species and disrupting redox systems, leading to cellular toxicity in cells (Yin et al., 2020). Saifi discovered that exposure pathways to Ag NPs can lead to the accumulation of toxic concentrations in the body, possibly resulting in organ toxicity, including impacts on the brain, liver, spleen, lymph nodes, and other organs (Saifi et al., 2018). Research into the toxicity of Cu NPs is currently limited. Carmona et al. reported a variety of adverse effects from Cu-NPs on mouse kidneys, livers, and spleens, both *in vitro* and *in vivo* experiments (Carmona et al., 2018). Sadiq et al. detected DNA strand breaks and oxidative DNA damage induced by Cu NPs, correlating cellular toxicity with released Cu ions (Sadiq et al., 2015). The low stability and aggregation of Cu NPs in aqueous solutions should be considered when interpreting their nanotoxicological effects.

Emerging research confirms that Ag-Cu NPs exhibit not only synergistically enhanced antibacterial efficacy but also a reduced potential for nanoparticle-induced bodily toxicity (Yin et al., 2020). Van Hengel believes that the synergistic effect of Ag-Cu NPs boosts antibacterial effectiveness while minimizing cytotoxicity by reducing the required Ag NPs (van Hengel et al., 2020). Długosz et al. contend that the reduced toxicity of Ag-Cu NPs primarily stems from their synergistic capacity to generate larger, less mobile nanoparticles. This inhibits the formation of detrimental free radicals and the binding process with -SH groups (Długosz et al., 2021). Further research has highlighted that in conventionally synthesized nanoparticles, aggregation serves as the primary source of toxicity. In Ag-Cu NPs, Ghadiri observed a more uniformly dispersed single nanoparticle, providing strong evidence of the safety performance of BNPs (Ghadiri et al., 2020). Furthermore, the genotoxicity of BNPs can be affected by various factors, encompassing aspects like their shape, surface characteristics, physicochemical parameters (such as pH and temperature), solubility, in addition to variables like the dosage of nanoparticles, duration of exposure, and the type of cells involved (Magdolenova et al., 2014). These findings hold significant implications for the future development of antimicrobial products.

## Outstanding attributes and influential factors in antibacterial domain

### Stability of BNPs

Silver nanoparticles (AgNPs) and copper nanoparticles (CuNPs) exhibit excellent antibacterial activity, along with higher safety and longer activity cycles compared to organic nanomaterials, even at low concentrations. Additionally, the susceptibility of CuNPs to oxidation in ambient aerobic conditions leads to the formation of irregularly shaped, often spherical, agglomerated particles during the preparation process, potentially compromising their physicochemical and antimicrobial performance (Dlugosz and Banach, 2020).

Relevant studies suggest that the incorporation of silver components significantly reduces the oxidation tendencies of CuNPs. The formation of Ag-Cu NPs reduces oxygen ingress, resulting in a more stable structure compared to monometallic conditions, allowing for slow ion release, both favorable for synergistic interactions between the two metals (Cruces et al., 2022). Ahmadinejad et al. investigated the aging of synthesized Ag-Cu alloy nanoparticles and found them to be more stable than pure Cu nanoparticles, attributing this stability to the protective effect of a thin Ag shell (Ahmadinejad and Mahdieh, 2022). Similarly, Tsai demonstrated, through experiments, that the formation temperature of Cu oxide in Ag-Cu NPs is at least 150°C higher than in similarly sized pure Cu nanoparticles, owing to the protective role of the thin Ag shell (Tsai et al., 2013).

The stability of nanoparticles is closely linked to their high surface-to-volume ratio and rapid, uncontrolled release characteristics. Ongoing research in the quest for long-term stable NPs includes Dlugosz et al.'s suggestion that the addition of higher concentrations of tannic acid during CuNPs synthesis favors the generation of more stable CuNPs products (Dlugosz and Banach, 2020). Cruces emphasizes the importance of carriers, suggesting that negatively charged surfaces of zeolite (Zeo) and montmorillonite (Mtt) facilitate a more uniform loading and even release of Ag-Cu NPs, significantly enhancing their stability (Cruces et al., 2022). Recently, Ahmadinejad discovered a correlation between the presence and intensity of an applied electric field and the size and stability of Ag-Cu alloy NPs. Under appropriate field strength, Ag-Cu NPs become increasingly stable with increasing field intensity, providing theoretical support for new development strategies (Ahmadinejad and Mahdieh, 2022). Furthermore, studies on the addition of stabilizers such as polyvinyl pyrrolidone (PVP)polyvinyl alcohol (PVA) have been reported to enhance stability (Zhu et al., 2021).

### Dispersion of BNPs

Numerous studies have substantiated the superior antibacterial capabilities of nanomaterials compared to their larger counterparts, attributed to their smaller size and higher dispersion. Nevertheless, the challenge of nanoparticle aggregation persists during synthesis. Kalinska attributes the aggregation tendency of Ag-Cu NPs to nanoparticle size heterogeneity (Kalińska et al., 2019), while Manikam and colleagues propose that high surface energy and thermodynamic instability are the primary aggregation causes (Manikam et al., 2011). Ghadiri argues that nanoparticle aggregation is an inherent outcome of their synthetic pathways. He posits that conventional methods of nanoparticle synthesis might induce substantial particle aggregation, consequently significantly enhancing their toxicity (Ghadiri et al., 2020). To maximize the antibacterial efficacy of Ag-Cu NPs and mitigate toxicity, it is crucial to employ diverse strategies to reduce nanoparticle aggregation.

Numerous strategies exist to enhance Ag-Cu NPs dispersion. Research findings highlight that precursor type, proportion, and addition order impact complex formation and nanoparticle dispersion. Furthermore, Ghorbi’s suggestion to moderate the acceleration rate of silver precursors has been shown to notably improve the dispersion of silver cores in CS NPs, effectively preventing aggregation in the resulting Ag-Cu NPs; however, surpassing an optimal precursor concentration may reduce nanoparticle dispersion and lead to increased cluster formation, adversely affecting overall performance (Ghorbi et al., 2019). Researchers stress the vital role of surfactants as essential stabilizers to prevent nanoparticle aggregation, while capping agents are emphasized for their potential to boost the antibacterial performance of nanoparticles and avert particle aggregation (Manikam et al., 2011; Joel T and Shobini, 2018). In addition to these points, Perdikaki’s research indicates that a significant increase in the surface area ratio of Ag-Cu NPs often reduces aggregation, enhancing antibacterial effects while lowering genetic toxicity (Perdikaki et al., 2016). Długosz further highlights that an increased proportion of Cu NPs results in Ag-Cu NPs with noticeably larger sizes than Ag NPs, effectively improving stability and dispersion while mitigating aggregation-related risks (Długosz et al., 2021).

### Dimensions of BNPs

Based on their geometric shapes, BNPs can be categorized into zero-dimensional NPs, one-dimensional NPs, and two-dimensional NPs. Zero-dimensional nanoparticles, such as nanospheres and polyhedra, are predominantly synthesized using wet-chemical methods. One-dimensional nanoparticles consist of nanowires, nanorods, and nanotubes, while two-dimensional nanoparticles are composed of nanoplates, nanosheets, and nanobelts. These BNPs exhibit distinct properties and catalytic performance depending on the available active sites (Duan and Wang, 2013).

Some researchers emphasize that the higher surface area ratio of BNPs is inherently linked to their smaller nano size. Smaller nanoparticle sizes are more favorable for the release of metal ions from regions rich in low-coordination atoms (Ghasemi et al., 2017; Sabira et al., 2020). S Simultaneously, the reduction in size significantly enhances the generation of reactive oxygen species (ROS), resulting in a more potent oxidative stress damage during the antimicrobial action of Ag-Cu NPs (Manke et al., 2013). These two synergistic mechanisms collectively enhance their antibacterial efficacy.

The study conducted by Dulgosz indicates that enhancing the ratio of Cu NPs leads to the formation of larger Ag-Cu NPs. This results in the creation of nanocomplexes characterized by improved stability and reduced toxicity (Długosz et al., 2021). Consequently, a substantial enhancement in bactericidal efficiency occurs, propelled by the advantageous interplay of synergistic effects—an insightful finding within their research (Długosz et al., 2021). However, Zhang presents a differing viewpoint, suggesting that the miscibility and orderliness of the bimetallic structure can be affected, potentially resulting in smaller BNPs compared to their individual NP counterparts. This increase in surface-to-volume ratio, accompanied by a higher number of active sites and lower energy barriers, significantly enhances the catalytic activity of BNPs (Zhang and Zhang, 2018). Furthermore, it is important to note that the presence of Ag-Cu NPs has a synergistic effect, reducing the requirement for Ag NPs and effectively mitigating the genotoxicity associated with BNPs (van Hengel et al., 2020).

### Impact of carrier presence on Ag-Cu NPs

**FIGURE 4:**
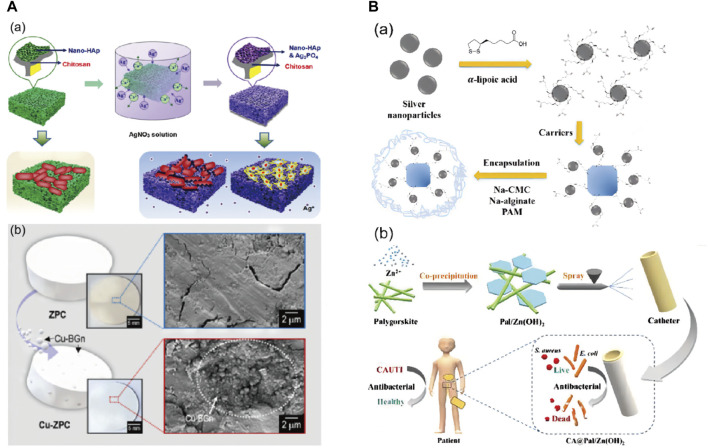
Classification of carriers for transporting nanomaterials according to their functions. **(A)**. Biocompatible carriers loaded with metal antibacterial active components; **(B)**. Adsorbable carriers loaded with metal antibacterial active components. (“A and B ” reprinted with permission from Ref (Yang et al., 2022).

As a significant constituent within loaded nanoparticles, the carrier has been extensively examined by numerous scholars. Montmorillonite (Roy et al., 2018), sepiolite (Li et al., 2020), nanofibers (Liu et al., 2018), carbon nanotubes (Jiang et al., 2022), carbon nanospheres (Li et al., 2016)), nano-SiO_**2**_ (Ermini and Voliani, 2021), and biopolymeric materials (Arfat et al., 2017b) have garnered attention as carriers (Table 3). Based on their functionality, Yang et al. categorized carriers into two main types: biocompatible (Figure 4A) and absorptive (Figure 4B). Among the biocompatible carriers are hydroxyapatite and bioactive glass, which mitigate immune responses upon introduction into organisms. Zeolite and clay minerals, classified as absorptive carriers, offer conducive environments for the loading and dispersion of inorganic nanometals (Yang et al., 2022). Yang et al. ascribed the antibacterial mechanism of the carriers to their sturdy pore structure and expansive specific surface area. These attributes facilitated the efficient absorption and release of active ingredients through diverse mechanisms, simultaneously playing a pivotal role in averting the agglomeration of high-surface-energy nanoparticles (Yang et al., 2022).

**TABLE 3:** Summarized applications and advantages of various nanoparticle carriers.

Nanocarriers	Carriers type	Nanomaterials	Advantages	References
MMT	Adsorbable types	Ag/MMT、Cu/MMT	A cost-effective and non-toxic clay nanomaterial, exhibits strong adsorption capabilities and stabilizes nanoparticles, preventing clumping	Roy et al. (2018)
		Ag/OMMT/QCS-QOMA		Chen et al. (2016b)
		(Ag-Nacre-like KGM)/MMT		Zhu et al. (2018)
		Ag/MMT		Costa et al. (2011)
		Ag/OMMT		Zhang et al. (2018b)
		Ag/MMT/Agar–CMC		Makwana et al. (2020)
		Ag/glassy/matrix/MMT		Esteban-Tejeda et al. (2010)
		Ag-Bi2O3/MMT		Tun et al. (2020)
Sep	Adsorbable types	Ag/Sep	Carrier Sep has abundant Si−OH ion clusters, ensuring strong adsorption, covalent bond formation, and enhancing thermal stability	Li et al. (2020)
		Si/Sep		Gómez-Avilés et al. (2013)
		MgO/Sep		Sidhu et al. (2020)
		TiO2/Sep		Bouna et al. (2011)
		Si-Al/Sep		Belver et al. (2013)
		ZnO/Fe3O4-SeP		Akkari et al. (2017)
Carbon Nanostructure	Adsorbable types	Ag/CNF	Leveraging carbon nanostructures for eco-friendly, non-toxic, highly biocompatible, degradable carriers, enhancing photocatalysis, with flexibility in size, shape, and surface properties	Liu et al. (2018)
		Ag-Pulp/CNF		Zhu et al. (2020b)
		Pd/SCNT-500		Jiang et al. (2022)
		G/ZnO_**2**_/CNF		Ahmadi et al. (2021)
		Ag/CNSs、Au/CNSs		Li et al. (2016)
HA	Adsorbable types	Zn/HA	HA possesses unique nanoscale properties, is widely sourced, non-toxic, with strong adsorption, significantly enhancing the surface area of loaded metal nanoparticles, and comes with inherent antibacterial properties	Murugesan et al. (2018)
		TiO2、Au、Pt/Chitosan/PLA/HA		Radwan-Pragłowska et al. (2020)
		Al_**2**_O_**3**_/HA		Ghosh et al. (2010)
		Fe_**3**_O_**4**_/HA		Singhal et al. (2017)
		Ag/HA		Qu et al. (2013)
Graphene	Adsorbable types	FeAg/Graphene	Graphene’s properties like large surface area, mechanical strength, electrical stability, and inherent antibacterial capacity significantly enhance nanocomposite activity and stabilize loaded nanoparticles	Ahmad et al. (2016)
		Au/Graphene		Chen et al. (2016a)
		Ag/RGO、Cu/RGO		Bhattacharjee et al. (2021)
		Au/Graphene		Barani et al. (2021)
		Au/Graphene		Bakhtiary et al. (2022)
Bioactive glass	Biocompatible types	Ce/BG	Biologically active materials, as carriers, possess therapeutic properties while effectively enhancing the dispersion of loaded nanoparticles, mitigating immune responses, and exhibiting excellent biocompatibility	Farag et al. (2019)
		γ-Fe2O3/BG		Kesse et al. (2020)
		CHT/BG-NPs		Correia et al. (2015)
		GelMA/BG		Mei et al. (2022)
Glycerol plasticized agar solution		Ag-Cu NPs		Arfat et al. (2017a)
Cur		Cu NPs、Ag NPs		Targhi et al. (2021)

Numerous studies have shown that Ag-Cu NPs with support have better activity than those without support (Ahmad et al., 2016). Perdikaki contends that the presence of carriers plays a crucial role in augmenting oxidative stress and cell membrane disruption by BNPs, while also ensuring the uniform distribution of nanoparticles on the surface (Perdikaki et al., 2016). Conversely, Targhi attributes the heightened antibacterial efficacy in the presence of carriers to the sustained-release effect exhibited by the nanoparticles. Carriers can ensure that nanoparticles maintain a more prolonged *in vitro* antibacterial and anti-biofilm effect at lower doses, thus eliciting a more enduring bactericidal action (Targhi et al., 2021). In addition, it has been well-documented that the partial carrier possesses intrinsic antibacterial capabilities and exhibits mechanisms that synergistically amplify oxidative stress damage in conjunction with nanoparticles, thereby enhancing their antibacterial effectiveness (Ahmad et al., 2016).

It is important to note that the application of most carrier materials is often constrained by various factors. These limitations include complex synthesis processes, unclear *in vivo* reaction mechanisms, and severe inflammation in vital organs such as the kidneys. Consequently, it is imperative to carefully evaluate the selection of carrier materials in future research, development, and application (Fan et al., 2020).

### pH of the external environment

Teixeira posits that pH can directly modulate the surface charge and electron transfer properties of BNPs, consequently influencing their photocatalytic capabilities and antibacterial attributes (Teixeira et al., 2019). Pelgrift et al., in their observation, found that the antibacterial potential of nanoparticles becomes readily activated in acidic conditions at the infection site (Pelgrift and Friedman, 2013). Further confirming this effect, Yan et al. demonstrated that BNPs exhibit increased bactericidal efficacy in a low pH environment (pH 5.0) compared to a physiological environment (pH 7.4). This enhancement is likely attributed to the substantial release of Cu^**2+**^ and Ag^**+**^ ions at low pH conditions (Yan et al., 2020).

### Optical and electrical properties of BNPs

Existing research has demonstrated that Cu NPs, a common p-type semiconductor, have gained widespread application in the fields of photocatalysis and sterilization due to their cost-effectiveness, unique physicochemical properties, high surface area, and promising prospects (Noman et al., 2020). Similarly, as a well-known active photocatalyst, Ag NPs possess tunable plasmonic resonance effects that can significantly mitigate infections caused by numerous bacteria (Asgari et al., 2022). While both Ag NPs and Cu NPs have their individual advantages in the field of photocatalysis, research on harnessing their synergistic effects for the synthesis of BNPs to enhance their antibacterial performance is scarce.

Scholars suggest that the photocatalytic antibacterial mechanism of hybrid semiconductor–metal nanoparticles (Ag-CuNPs) relies on charge separation and transfer between the metal and semiconductor materials (Kushwah et al., 2019). This charge separation was substantiated by Waiskopf et al. through the observation of fluorescence quenching effects in HNPs (Waiskopf et al., 2018). Therefore, the heightened bactericidal effect of Ag-Cu nanoparticles may be linked to the charge transfer mechanism. Panchal discovered that HNPs exhibited higher hydroxyl radical concentrations under light, thanks to electrons transferring from semiconductors to metal nanoparticles (Panchal et al., 2020) Similarly, Ag NPs with surface plasmon resonance released electrons to interact with oxygen, producing additional ROS. These amplified free radicals and ROS bolstered the oxidative stress response of Ag-Cu NPs (Zhu M. et al., 2020).

Various factors influence charge separation and hole removal between HNPs particles. Ben-Shahar indicated that surface and chemical properties of the surroundings impact HNPs charge separation. They linked charge separation to HNPs size, with a denser state-of-states in metal compounds boosting transfer rates for larger metal domains (Ben-Shahar et al., 2016). Waiskopf suggested that the metal composition plays a role in influencing charge transfer kinetics and efficiency. It was observed that more efficient charge transfer occurs in semiconductor nanorods modified with multi-island structures (Waiskopf et al., 2018). Moreover, it has been documented in the literature that environmental variables, including the presence of organic ligands and highly alkaline conditions, significantly enhance the process of charge separation (Simon et al., 2014).

### Oxidative stress response

**FIGURE 5:**
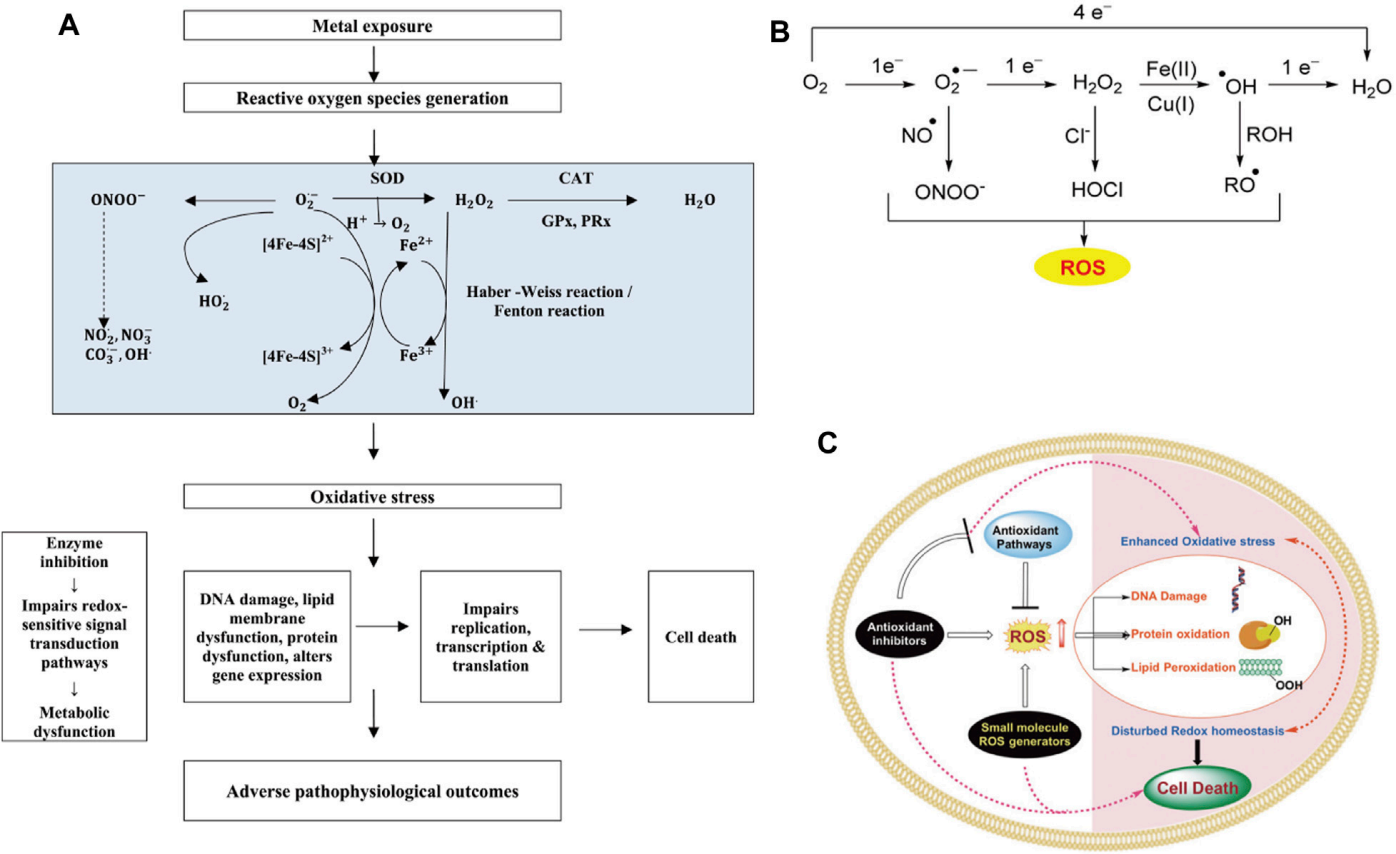
Generation and function of oxidative stress response. **(A)** Metal-induced oxidative stress-mediated adverse pathophysiological outcome (Khalid et al., 2020). **(B)** Sources of oxidative stress for bacteria. **(C)** The potential mechanism of reactive oxygen species (ROS) in antibacterial action. (“B and C″ reprinted with permission from (Dharmaraja, 2017). Copyright 2017 American Chemical Society).

Reactive oxygen species (ROS) are highly active oxygen-containing molecules, including unstable radicals like superoxide anions, hydroxyl radicals, and hydrogen peroxide. Research has demonstrated that cells can generate reactive oxygen species (ROS) through both endogenous pathways, such as NADPH oxidase, and exogenous pathways, including metal-catalyzed Fenton reactions (Manke et al., 2013). After exposure to nanoparticles (NPs), Dharmaraja observed an elevation in reactive oxygen species (ROS) production within microbial cells, resulting in oxidative damage to biomacromolecules such as lipids, proteins, and nucleic acids (Dharmaraja, 2017). The generation mechanism and bactericidal action of ROS are illustrated in Figure 5.

Manke has advanced the idea that when faced with an overabundance of ROS generation, host cells employ a regulatory mechanism involving the expression of cytokines (MAPK, PTPs, Src, NF-𝜅B, AP-1) to mitigate oxidative stress-induced genotoxicity. This regulation is facilitated by the coordinated action of non-enzymatic antioxidants, including enzymes (SOD, CAT, PER), as well as other substances (Vc, VE, GSH, Cys) (Manke et al., 2013).

Previous studies have shown that compared to other metal nanoparticles, Ag NPs and Cu NPs tend to generate a richer pool of ROS, consequently leading to more pronounced oxidative stress damage (Długosz et al., 2021). Meghana discovered that under aerobic conditions, Cu NPs not only rapidly degrade cell membranes but also generate extremely high concentrations of ROS. This ROS-induced oxidative stress damage serves as one of the primary mechanisms behind the antibacterial properties of copper nanoparticles (Meghana et al., 2015). Liao et al. proposed that the oxidative stress bactericidal mechanism of Ag NPs is linked to the significant inhibition of redox-related enzymes in bacterial cells, such as CAT and POD (Liao et al., 2019). Recently, Bondarenko identified a relatively novel phenomenon. He observed a strong correlation between the dissolution rate of Ag NPs and their primary bactericidal mechanism and toxic effects. Ag NPs with a high dissolution rate tend to primarily employ the bactericidal mechanism through the release of Ag^**+**^. In contrast, Ag NPs with a low dissolution rate demonstrate a greater tendency to induce ROS-mediated damage and exhibit minimal genotoxicity (Bondarenko et al., 2018).

Emerging research highlights the potent synergistic effect of Ag-Cu NPs, significantly elevating ROS levels within microbial systems, influenced by a variety of factors. Długosz observed that BNPs can enhance their antibacterial performance by increasing their surface-to-volume ratio, accelerating ion release, and intensifying ROS content and oxidative stress reactions (Długosz et al., 2021). Ahmad et al. emphasized the significance of carriers in ROS generation within BNPs systems (Ahmad et al., 2016; Targhi et al., 2021). Additionally, Metryka suggested that the presence of transition metals (Cu NPs) on the surface of BNPs contributes to the generation of additional ROS through Fenton, Fenton-like reactions, and the Haber-Weiss reaction, broadening our understanding of the multifaceted antibacterial mechanisms of NPs (Metryka et al., 2021).

The impact of the external environment should not be underestimated. Moussa proposed that under illuminated conditions, photo-excited charge-carrier interactions in the Fenton reaction enhance ROS levels within bacterial cells, facilitating BNPs’ effective oxidative stress bactericidal activity (Moussa et al., 2016). Zhang et al. emphasized the indispensable role of oxygen in oxidative stress reactions within the BNPs system (Zhang et al., 2017). André suggested that the reduced activity of BNPs in anaerobic environments primarily stems from decreased macrophage and neutrophil activity (André et al., 2022). Additionally, the influence of pH levels and temperature conditions in the surrounding environment on the oxidative stress damage caused by Ag-Cu NPs has been substantiated by relevant scholars (Sun et al., 2021; Hosny et al., 2022), offering insights for optimizing the antibacterial effectiveness of NPs.

## Conclusion and outlook

In conclusion, Ag-Cu NPs exhibit remarkable bactericidal efficacy and minimal genotoxicity compared to their individual nanoparticle counterparts. The incorporation of BNPs not only addresses the inherent aggregation susceptibility of Cu NPs, but also mitigates the significant genotoxic effects associated with Ag NPs, thereby optimizing the beneficial attributes of single nanoparticle species. Moreover, the synergistic mechanisms intrinsic to BNPs facilitate a substantial elevation of ROS levels, with robust oxidative stress damage emerging as the primary bactericidal mechanism. The performance of bimetallic nanoparticles is significantly modulated by external factors such as pH, carrier presence, oxygen levels, and light conditions. For potential clinical applications, the pursuit of enhanced BNPs performance undoubtedly represents a burgeoning Frontier with considerable promise.

Past research has predominantly employed conventional methods to synthesize Ag-Cu NPs, which are expensive, hazardous, and time-consuming. Hence, green synthesis presents an appealing alternative for creating non-toxic, cost-effective, and environmentally friendly metal nanoparticles. In this approach, unicellular and multicellular organisms like microorganisms and plants are utilized for nanoparticle synthesis. Despite the lower cost, improved efficacy, smaller particle sizes, and environmentally friendly bio-compatibility associated with green synthesis, its limitations cannot be overlooked. Challenges include uneven dispersion of NPs, slow production rates, and difficulties in achieving precise control over size distribution, shape, and crystallinity. These factors make the implementation of these biological methods challenging for large-scale production. Future endeavors may focus on exploring bio-synthesis methods to overcome the constraints of microbial and plant-based preparation routes. Simplicity, safety, absence of toxic chemicals, enhanced stability, and improved physical and chemical properties are key factors when considering the green synthesis method.

Research has demonstrated the critical influence of the carrier on the physical properties of Ag-Cu NPs. Current studies on the clinical application of nanocarriers mainly focus on the loading and dispersion of nanoparticles, drug release, and delivery mechanisms. Therefore, the assessment of nanocarrier’s hepatorenal toxicity has become increasingly important during application. Future research may focus on the development of nanocarriers with enhanced safety features, such as biodegradable nanocarriers or naturally derived carriers from plants (e.g., curcumin, humic acid). Additionally, to enhance the antimicrobial effects of nanocomposites, research into carriers with intrinsic antimicrobial properties could emerge as a current research focus.

Moving forward, gaining a deeper understanding of novel mechanisms is essential for synthesizing unique metal nanocomposites and exploring their potential applications. The synthesis of inorganic metal nanocomposites with stronger bactericidal ability and no toxicity is desired for clinical trials. Scalable applications from laboratory to commercial scale are also valuable. This review summarizes the bactericidal mechanism and influencing factors of silver and copper nano complexes and their oxides, providing clues for future research in this important field.

## References

[B1] Abbasi B. H. Zaka M. Hashmi S. S. Khan Z. (2018). Biogenic synthesis of Au, Ag and Au–Ag alloy nanoparticles using *Cannabis sativa* leaf extract. IET nanobiotechnol 12, 277–284. 10.1049/iet-nbt.2017.0169

[B2] Agnihotri R. Gaur S. Albin S. (2020). Nanometals in dentistry: applications and toxicological implications-a systematic review. Biol. Trace Elem. Res. 197, 70–88. 10.1007/s12011-019-01986-y 31782063

[B3] Ahmad A. Qureshi A. S. Li L. Bao J. Jia X. Xu Y. (2016). Antibacterial activity of graphene supported FeAg bimetallic nanocomposites. Colloids Surf. B Biointerfaces 143, 490–498. 10.1016/j.colsurfb.2016.03.065 27038914

[B4] Ahmadi A. Ahmadi P. Sani M. A. Ehsani A. Ghanbarzadeh B. (2021). Functional biocompatible nanocomposite films consisting of selenium and zinc oxide nanoparticles embedded in gelatin/cellulose nanofiber matrices. Int. J. Biol. Macromol. 175, 87–97. 10.1016/j.ijbiomac.2021.01.135 33485892

[B5] Ahmadinejad E. Mahdieh M. H. (2022). Laser-assisted synthesis of Ag–Cu alloy nanoparticles with tunable surface plasmon resonance frequency in presence of external electric field. J. Laser Appl. 34, 012004. 10.2351/7.0000535

[B6] Akkari M. Aranda P. Mayoral A. García-Hernández M. Ben Haj Amara A. Ruiz-Hitzky E. (2017). Sepiolite nanoplatform for the simultaneous assembly of magnetite and zinc oxide nanoparticles as photocatalyst for improving removal of organic pollutants. J. Hazard. Mater. 340, 281–290. 10.1016/j.jhazmat.2017.06.067 28715751

[B7] Alavi M. Karimi N. (2018a). Antiplanktonic, antibiofilm, antiswarming motility and anti*quorum* sensing activities of green synthesized Ag-TiO2, TiO2-Ag, Ag-Cu and Cu-Ag nanocomposites against multi-drug-resistant bacteria. Artif. Cells Nanomed Biotechnol. 46, S399–S413. 10.1080/21691401.2018.1496923 30095025

[B8] Alavi M. Karimi N. (2018b). Characterization, antibacterial, total antioxidant, scavenging, reducing power and ion chelating activities of green synthesized silver, copper and titanium dioxide nanoparticles using Artemisia haussknechtii leaf extract. Artif. Cells Nanomed Biotechnol. 46, 2066–2081. 10.1080/21691401.2017.1408121 29233039

[B9] Almutairi H. H. Parveen N. Ansari S. A. (2022). Hydrothermal synthesis of multifunctional bimetallic Ag-CuO nanohybrids and their antimicrobial, antibiofilm and antiproliferative potential. Nanomater. (Basel) 12, 4167. 10.3390/nano12234167 PMC9737815 36500789

[B10] Ameen F. (2022). Optimization of the synthesis of fungus-mediated Bi-metallic Ag-Cu nanoparticles. Appl. Sci. 12, 1384. 10.3390/app12031384

[B11] André A. C. Laborde M. Marteyn B. S. (2022). The battle for oxygen during bacterial and fungal infections. Trends Microbiol. 30, 643–653. 10.1016/j.tim.2022.01.002 35131160

[B12] Arfat Y. A. Ahmed J. Jacob H. (2017a). Preparation and characterization of agar-based nanocomposite films reinforced with bimetallic (Ag-Cu) alloy nanoparticles. Carbohydr. Polym. 155, 382–390. 10.1016/j.carbpol.2016.08.097 27702525

[B13] Arfat Y. A. Ejaz M. Jacob H. Ahmed J. (2017b). Deciphering the potential of guar gum/Ag-Cu nanocomposite films as an active food packaging material. Carbohydr. Polym. 157, 65–71. 10.1016/j.carbpol.2016.09.069 27987974

[B14] Arora N. Thangavelu K. Karanikolos G. N. (2020). Bimetallic nanoparticles for antimicrobial applications. Front. Chem. 8, 412. 10.3389/fchem.2020.00412 32671014 PMC7326054

[B15] Asgari S. Mohammadi Ziarani G. Badiei A. Setayeshmehr M. Kiani M. Pourjavadi A. (2022). Electrospun Ag-decorated reduced GO-graft-chitosan composite nanofibers with visible light photocatalytic activity for antibacterial performance. Chemosphere 299, 134436. 10.1016/j.chemosphere.2022.134436 35358565

[B16] Bakhtiary S. Chegeni A. Babaeipour V. Omidi M. Keshel S. H. Khodamoradi N. (2022). Culture and maintenance of neural progressive cells on cellulose acetate/graphene-gold nanocomposites. Int. J. Biol. Macromol. 210, 63–75. 10.1016/j.ijbiomac.2022.05.026 35537583

[B17] Barani M. Mukhtar M. Rahdar A. Sargazi G. Thysiadou A. Kyzas G. Z. (2021). Progress in the application of nanoparticles and graphene as drug carriers and on the diagnosis of brain infections. Molecules 26, 186. 10.3390/molecules26010186 33401658 PMC7795866

[B18] Barnes C. A. Elsaesser A. Arkusz J. Smok A. Palus J. Leśniak A. (2008). Reproducible comet assay of amorphous silica nanoparticles detects no genotoxicity. Nano Lett. 8, 3069–3074. 10.1021/nl801661w 18698730

[B19] Behera A. Mittu B. Padhi S. Patra N. Singh J. (2020). “Bimetallic nanoparticles: green synthesis, applications, and future perspectives,” in Multifunctional hybrid nanomaterials for sustainable agri-food and ecosystems (Amsterdam, Netherlands: Elsevier), 639–682. 10.1016/B978-0-12-821354-4.00025-X

[B20] Belver C. Aranda P. Ruiz-Hitzky E. (2013). Silica–alumina/sepiolite nanoarchitectures. J. Mat. Chem. A 1, 7477. 10.1039/c3ta01686b 23763177

[B21] Benetti G. Cavaliere E. Brescia R. Salassi S. Ferrando R. Vantomme A. (2019). Tailored Ag-Cu-Mg multielemental nanoparticles for wide-spectrum antibacterial coating. Nanoscale 11, 1626–1635. 10.1039/c8nr08375d 30644952

[B22] Ben-Shahar Y. Scotognella F. Kriegel I. Moretti L. Cerullo G. Rabani E. (2016). Optimal metal domain size for photocatalysis with hybrid semiconductor-metal nanorods. Nat. Commun. 7, 10413. 10.1038/ncomms10413 26783194 PMC4735686

[B23] Bhattacharjee R. Kumar L. Mukerjee N. Anand U. Dhasmana A. Preetam S. (2022). The emergence of metal oxide nanoparticles (NPs) as a phytomedicine: a two-facet role in plant growth, nano-toxicity and anti-phyto-microbial activity. Biomed. Pharmacother. 155, 113658. 10.1016/j.biopha.2022.113658 36162370

[B24] Bhattacharjee S. Joshi R. Yasir M. Adhikari A. Chughtai A. A. Heslop D. (2021). Graphene- and nanoparticle-embedded antimicrobial and biocompatible cotton/silk fabrics for protective clothing. ACS Appl. Bio Mater 4, 6175–6185. 10.1021/acsabm.1c00508 35006896

[B25] Bhol P. Bhavya M. B. Swain S. Saxena M. Samal A. K. (2020). Modern chemical routes for the controlled synthesis of anisotropic bimetallic nanostructures and their application in catalysis. Front. Chem. 8, 357. 10.3389/fchem.2020.00357 32528924 PMC7262677

[B26] Boas D. Remennik S. Reches M. (2023). Peptide-capped Au and Ag nanoparticles: detection of heavy metals and photochemical core/shell formation. J. Colloid Interface Sci. 631, 66–76. 10.1016/j.jcis.2022.10.154 36371827

[B27] Bogatyrenko S. I. Kryshtal A. P. Kruk A. (2023). Effect of size on the formation of solid solutions in Ag-Cu nanoparticles. J. Phys. Chem. C Nanomater Interfaces 127, 2569–2580. 10.1021/acs.jpcc.2c07132 36818666 PMC9931174

[B28] Bondarenko O. Ivask A. Käkinen A. Kurvet I. Kahru A. (2013). Particle-cell contact enhances antibacterial activity of silver nanoparticles. PLoS One 8, e64060. 10.1371/journal.pone.0064060 23737965 PMC3667828

[B29] Bondarenko O. M. Sihtmäe M. Kuzmičiova J. Ragelienė L. Kahru A. Daugelavičius R. (2018). Plasma membrane is the target of rapid antibacterial action of silver nanoparticles in *Escherichia coli* and *Pseudomonas aeruginosa* . Int. J. Nanomedicine 13, 6779–6790. 10.2147/IJN.S177163 30498344 PMC6207270

[B30] Bouazizi N. Bargougui R. Thebault P. Clamens T. Desriac F. Fioresi F. (2018). Development of a novel functional core-shell-shell nanoparticles: from design to anti-bacterial applications. J. Colloid Interface Sci. 513, 726–735. 10.1016/j.jcis.2017.11.074 29220687

[B31] Bouna L. Rhouta B. Amjoud M. Maury F. Lafont M.-C. Jada A. (2011). Synthesis, characterization and photocatalytic activity of TiO2 supported natural palygorskite microfibers. Appl. Clay Sci. 52, 301–311. 10.1016/j.clay.2011.03.009

[B32] Carmona E. R. García-Rodríguez A. Marcos R. (2018). Genotoxicity of copper and nickel nanoparticles in somatic cells of *Drosophila melanogaster* . J. Toxicol. 2018, 1–8. 10.1155/2018/7278036 PMC6077325 30111998

[B33] Caruso F. (2001). Nanoengineering of particle surfaces. Adv. Mat. 13, 11–22. 10.1002/1521-4095(200101)13:1<11::AID-ADMA11>3.0.CO;2-N

[B34] Chen C. Li N. Lan J. Ji X. He Z. (2016a). A label-free colorimetric platform for DNA via target-catalyzed hairpin assembly and the peroxidase-like catalytic of graphene/Au-NPs hybrids. Anal. Chim. Acta 902, 154–159. 10.1016/j.aca.2015.10.030 26703265

[B35] Chen K. Ye W. Cai S. Huang L. Zhong T. Chen L. (2016b). Green antimicrobial coating based on quaternised chitosan/organic montmorillonite/Ag NPs nanocomposites. J. Exp. Nanosci. 11, 1360–1371. 10.1080/17458080.2016.1227095

[B36] Chen Z. Mochizuki D. Maitani M. M. Wada Y. (2013). Facile synthesis of bimetallic Cu–Ag nanoparticles under microwave irradiation and their oxidation resistance. Nanotechnology 24, 265602. 10.1088/0957-4484/24/26/265602 23732107

[B37] Correia C. O. Leite Á. J. Mano J. F. (2015). Chitosan/bioactive glass nanoparticles scaffolds with shape memory properties. Carbohydr. Polym. 123, 39–45. 10.1016/j.carbpol.2014.12.076 25843832

[B38] Costa C. Conte A. Buonocore G. G. Del Nobile M. A. (2011). Antimicrobial silver-montmorillonite nanoparticles to prolong the shelf life of fresh fruit salad. Int. J. Food Microbiol. 148, 164–167. 10.1016/j.ijfoodmicro.2011.05.018 21684619

[B39] Cruces E. Arancibia-Miranda N. Manquian-Cerda K. Perreault F. Bolan N. Ignacio Azocar M. (2022). Copper/silver bimetallic nanoparticles supported on aluminosilicate geomaterials as antibacterial agents. ACS Appl. Nano Mat. 5, 1472–1483. 10.1021/acsanm.1c04031

[B40] Cybula A. Priebe J. B. Pohl M.-M. Sobczak J. W. Schneider M. Zielińska-Jurek A. (2014). The effect of calcination temperature on structure and photocatalytic properties of Au/Pd nanoparticles supported on TiO2. Appl. Catal. B Environ. 152–153, 202–211. 10.1016/j.apcatb.2014.01.042

[B41] Dehghani S. Peighambardoust S. H. Peighambardoust S. J. Hosseini S. V. Regenstein J. M. (2019). Improved mechanical and antibacterial properties of active LDPE films prepared with combination of Ag, ZnO and CuO nanoparticles. Food Packag. Shelf Life 22, 100391. 10.1016/j.fpsl.2019.100391

[B42] Delsante S. Borzone G. Novakovic R. Piazza D. Pigozzi G. Janczak-Rusch J. (2015). Synthesis and thermodynamics of Ag-Cu nanoparticles. Phys. Chem. Chem. Phys. 17, 28387–28393. 10.1039/c5cp02058a 26112754

[B43] Dharmaraja A. T. (2017). Role of reactive oxygen species (ROS) in therapeutics and drug resistance in cancer and bacteria. J. Med. Chem. 60, 3221–3240. 10.1021/acs.jmedchem.6b01243 28135088

[B44] Dlamini N. G. Basson A. K. Pullabhotla V. S. R. (2023). Synthesis and characterization of various bimetallic nanoparticles and their application. Appl. Nano 4, 1–24. 10.3390/applnano4010001

[B45] Dlugosz O. Banach M. (2020). Continuous synthesis of metal and metal oxide nanoparticles in microwave reactor. Colloid Surf. a-physicochem. Eng. Asp. 606, 125453. 10.1016/j.colsurfa.2020.125453

[B46] Długosz O. Sochocka M. Ochnik M. Banach M. (2021). Metal and bimetallic nanoparticles: flow synthesis, bioactivity and toxicity. J. Colloid Interface Sci. 586, 807–818. 10.1016/j.jcis.2020.11.005 33198985

[B47] Duan S. Wang R. (2013). Bimetallic nanostructures with magnetic and noble metals and their physicochemical applications. Prog. Nat. Sci. Mater. Int. 23, 113–126. 10.1016/j.pnsc.2013.02.001

[B48] Egger S. Lehmann R. P. Height M. J. Loessner M. J. Schuppler M. (2009). Antimicrobial properties of a novel silver-silica nanocomposite material. Appl. Environ. Microbiol. 75, 2973–2976. 10.1128/AEM.01658-08 19270121 PMC2681698

[B49] Ermini M. L. Voliani V. (2021). Antimicrobial nano-agents: the copper age. ACS Nano 15, 6008–6029. 10.1021/acsnano.0c10756 33792292 PMC8155324

[B50] Esteban‐Tejeda L. Malpartida F. Pecharromán C. Moya J. S. (2010). High antibacterial and antifungal activity of silver monodispersed nanoparticles embedded in a glassy matrix. Adv. Eng. Mater 12. 10.1002/adem.200980077

[B51] Fan Z. Wang Y. Xiang S. Zuo W. Huang D. Jiang B. (2020). Dual-self-recognizing, stimulus-responsive and carrier-free methotrexate-mannose conjugate nanoparticles with highly synergistic chemotherapeutic effects. J. Mater Chem. B 8, 1922–1934. 10.1039/d0tb00049c 32052817

[B52] Fanoro O. T. Oluwafemi O. S. (2020). Bactericidal antibacterial mechanism of plant synthesized silver, gold and bimetallic nanoparticles. Pharmaceutics 12, 1044. 10.3390/pharmaceutics12111044 33143388 PMC7693967

[B53] Farag M. M. Al-Rashidy Z. M. Ahmed M. M. (2019). *In vitro* drug release behavior of Ce-doped nano-bioactive glass carriers under oxidative stress. J. Mater Sci. Mater Med. 30, 18. 10.1007/s10856-019-6220-3 30671708

[B54] Fazio E. Gökce B. De Giacomo A. Meneghetti M. Compagnini G. Tommasini M. (2020). Nanoparticles engineering by pulsed laser ablation in liquids: concepts and applications. Nanomater. (Basel) 10, 2317. 10.3390/nano10112317 PMC7700616 33238455

[B55] Feng H. Wang W. Wang W. Zhang M. Wang C. Ma C. (2021). Charge transfer channels of silver @ cuprous oxide heterostructure core-shell nanoparticles strengthen high photocatalytic antibacterial activity. J. Colloid Interface Sci. 601, 531–543. 10.1016/j.jcis.2021.05.113 34090030

[B56] Ferrando R. Jellinek J. Johnston R. L. (2008). Nanoalloys: from theory to applications of alloy clusters and nanoparticles. Chem. Rev. 108, 845–910. 10.1021/cr040090g 18335972

[B57] Gan T. Wang Z. Shi Z. Zheng D. Sun J. Liu Y. (2018). Graphene oxide reinforced core-shell structured Ag@Cu2O with tunable hierarchical morphologies and their morphology-dependent electrocatalytic properties for bio-sensing applications. Biosens. Bioelectron. 112, 23–30. 10.1016/j.bios.2018.04.029 29689501

[B58] Garza-Cervantes J. A. Chávez-Reyes A. Castillo E. C. García-Rivas G. Antonio Ortega-Rivera O. Salinas E. (2017). Synergistic antimicrobial effects of silver/transition-metal combinatorial treatments. Sci. Rep. 7, 903. 10.1038/s41598-017-01017-7 28420878 PMC5429853

[B59] Ghadiri A. M. Rabiee N. Bagherzadeh M. Kiani M. Fatahi Y. Di Bartolomeo A. (2020). Green synthesis of CuO- and Cu2O-NPs in assistance with high-gravity: the flowering of nanobiotechnology. Nanotechnology 31, 425101. 10.1088/1361-6528/aba142 32604076

[B60] Ghasemi N. Jamali-Sheini F. Zekavati R. (2017). CuO and Ag/CuO nanoparticles: biosynthesis and antibacterial properties. Mater. Lett. 196, 78–82. 10.1016/j.matlet.2017.02.111

[B61] Ghorbi E. Namavar M. Rashedi V. Farhadinejad S. Pilban Jahromi S. Zareian M. (2019). Influence of nano-copper oxide concentration on bactericidal properties of silver–copper oxide nanocomposite. Colloids Surfaces A Physicochem. Eng. Aspects 580, 123732. 10.1016/j.colsurfa.2019.123732

[B62] Ghosh M. Mandal S. Roy A. Paladhi A. Mondal P. Hira S. K. (2021a). Synthesis and characterization of a novel drug conjugated copper-silver- titanium oxide nanocomposite with enhanced antibacterial activity. J. Drug Deliv. Sci. Technol. 62, 102384. 10.1016/j.jddst.2021.102384

[B63] Ghosh S. Ahmad R. Zeyaullah Md. Khare S. K. (2021b). Microbial nano-factories: synthesis and biomedical applications. Front. Chem. 9, 626834. 10.3389/fchem.2021.626834 33937188 PMC8085502

[B64] Ghosh S. Mashayekhi H. Bhowmik P. Xing B. (2010). Colloidal stability of Al _2_ O _3_ nanoparticles as affected by coating of structurally different humic acids. Langmuir 26, 873–879. 10.1021/la902327q 19813721

[B65] Ghosh Chaudhuri R. Paria S. (2012). Core/shell nanoparticles: classes, properties, synthesis mechanisms, characterization, and applications. Chem. Rev. 112, 2373–2433. 10.1021/cr100449n 22204603

[B66] Giorgetti E. Marsili P. Canton P. Muniz-Miranda M. Caporali S. Giammanco F. (2013). Cu/Ag-based bifunctional nanoparticles obtained by one-pot laser-assisted galvanic replacement. J. Nanopart. Res. 15, 1360. 10.1007/s11051-012-1360-0

[B67] Gómez-Avilés A. Aranda P. Fernandes F. M. Belver C. Ruiz-Hitzky E. (2013). Silica-sepiolite nanoarchitectures. J. Nanosci. Nanotechnol. 13, 2897–2907. 10.1166/jnn.2013.7429 23763177

[B68] Gu H. Chen X. Chen F. Zhou X. Parsaee Z. (2018). Ultrasound-assisted biosynthesis of CuO-NPs using brown alga Cystoseira trinodis: characterization, photocatalytic AOP, DPPH scavenging and antibacterial investigations. Ultrason. Sonochem 41, 109–119. 10.1016/j.ultsonch.2017.09.006 29137732

[B69] Hamouda R. A. Alharthi M. A. Alotaibi A. S. Alenzi A. M. Albalawi D. A. Makharita R. R. (2023). Biogenic nanoparticles silver and copper and their composites derived from marine alga *Ulva lactuca*: insight into the characterizations, antibacterial activity, and anti-biofilm formation. Molecules 28, 6324. 10.3390/molecules28176324 37687153 PMC10489668

[B70] Hosny M. Fawzy M. Eltaweil A. S. (2022). Phytofabrication of bimetallic silver-copper/biochar nanocomposite for environmental and medical applications. J. Environ. Manag. 316, 115238. 10.1016/j.jenvman.2022.115238 35576706

[B71] Jang J. Lee J.-M. Oh S.-B. Choi Y. Jung H.-S. Choi J. (2020). Development of antibiofilm nanocomposites: Ag/Cu bimetallic nanoparticles synthesized on the surface of graphene oxide nanosheets. ACS Appl. Mater Interfaces 12, 35826–35834. 10.1021/acsami.0c06054 32667802

[B72] Jiang H. Zang C. Guo L. Gao X. (2022). Carbon vacancies enriched carbon nitride nanotubes for Pd coordination environment optimization: highly efficient photocatalytic hydrodechlorination and CO2 cycloaddition. Sci. Total Environ. 838, 155920. 10.1016/j.scitotenv.2022.155920 35588820

[B73] Joel T J. Shobini J. (2018). A plausible antibacterial green synthesized AgNPs from tridax procumbens leaf-flower extract. J. Pure Appl. Microbiol. 12, 2135–2142. 10.22207/JPAM.12.4.51

[B74] Kalińska A. Jaworski S. Wierzbicki M. Gołębiewski M. (2019). Silver and copper nanoparticles—an alternative in future mastitis treatment and prevention? IJMS 20, 1672. 10.3390/ijms20071672 30987188 PMC6480535

[B75] Kesse X. Adam A. Begin-Colin S. Mertz D. Larquet E. Gacoin T. (2020). Elaboration of superparamagnetic and bioactive multicore-shell nanoparticles (γ-Fe2O3@SiO2-CaO): a promising material for bone cancer treatment. ACS Appl. Mater Interfaces 12, 47820–47830. 10.1021/acsami.0c12769 32990423

[B76] Khalid M. Hassani S. Abdollahi M. (2020). Metal-induced oxidative stress: an evidence-based update of advantages and disadvantages. Curr. Opin. Toxicol. 20 (21), 55–68. 10.1016/j.cotox.2020.05.006

[B77] Kumar P. Sharma P. K. Chaturvedi S. Singh S. Tripathi S. Fatima H. (2023). Green production of biologically active Ag and Ag-Cu nanoparticles from *Prosopis cineraria* pod waste extract and their application in epoxidation. Res. Chem. Intermed. 49, 557–575. 10.1007/s11164-022-04887-3

[B78] Kurt Yilmaz N. Schiffer C. A. (2021). Introduction: drug resistance. Chem. Rev. 121, 3235–3237. 10.1021/acs.chemrev.1c00118 33757288 PMC8164520

[B79] Kushwah M. Gaur M. S. Berlina A. N. Arora K. (2019). Biosynthesis of novel Ag@Cu alloy NPs for enhancement of methylene blue photocatalytic activity and antibacterial activity. Mat. Res. Express 6, 116561. 10.1088/2053-1591/ab485e

[B80] Lee C. Kim N. R. Koo J. Lee Y. J. Lee H. M. (2015). Cu-Ag core–shell nanoparticles with enhanced oxidation stability for printed electronics. Nanotechnology 26, 455601. 10.1088/0957-4484/26/45/455601 26489391

[B81] Li D. Gao X. Huang X. Liu P. Xiong W. Wu S. (2020). Preparation of organic-inorganic chitosan@silver/sepiolite composites with high synergistic antibacterial activity and stability. Carbohydr. Polym. 249, 116858. 10.1016/j.carbpol.2020.116858 32933687

[B82] Li L. Feng D. Zhang Y. (2016). Simultaneous detection of two tumor markers using silver and gold nanoparticles decorated carbon nanospheres as labels. Anal. Biochem. 505, 59–65. 10.1016/j.ab.2016.04.014 27156810

[B83] Li R. Wu N. Liu J. Jin Y. Chen X.-B. Zhang T. (2017). Formation and evolution of nanoporous bimetallic Ag-Cu alloy by electrochemically dealloying Mg-(Ag-Cu)-Y metallic glass. Corros. Sci. 119, 23–32. 10.1016/j.corsci.2017.02.017

[B84] Li X. Fu L. Ouyang J. Yang H. (2014). Microwave-assisted synthesis and interfacial features of CdS/kaolinite nanocomposite. Colloids Surfaces A Physicochem. Eng. Aspects 443, 72–79. 10.1016/j.colsurfa.2013.10.054

[B85] Liao S. Zhang Y. Pan X. Zhu F. Jiang C. Liu Q. (2019). <p&gt;Antibacterial activity and mechanism of silver nanoparticles against multidrug-resistant <em&gt;*Pseudomonas aeruginosa*&lt;/em&gt;</p&gt;. Int. J. Nanomedicine 14, 1469–1487. 10.2147/IJN.S191340 30880959 PMC6396885

[B86] Liu R. Dai L. Si C. Zeng Z. (2018). Antibacterial and hemostatic hydrogel via nanocomposite from cellulose nanofibers. Carbohydr. Polym. 195, 63–70. 10.1016/j.carbpol.2018.04.085 29805020

[B87] Liu X. Du J. Shao Y. Zhao S.-F. Yao K.-F. (2017). One-pot preparation of nanoporous Ag-Cu@Ag core-shell alloy with enhanced oxidative stability and robust antibacterial activity. Sci. Rep. 7, 10249. 10.1038/s41598-017-10630-5 28860477 PMC5579282

[B88] Ma W. Mashimo T. Tamura S. Tokuda M. Yoda S. Tsushida M. (2020). Cerium oxide (CeO2-x) nanoparticles with high Ce3+ proportion synthesized by pulsed plasma in liquid. Ceram. Int. 46, 26502–26510. 10.1016/j.ceramint.2020.07.093

[B89] Magdolenova Z. Collins A. Kumar A. Dhawan A. Stone V. Dusinska M. (2014). Mechanisms of genotoxicity. A review of *in vitro* and *in vivo* studies with engineered nanoparticles. Nanotoxicology 8, 233–278. 10.3109/17435390.2013.773464 23379603

[B90] Mahara Y. Ishikawa H. Ohyama J. Sawabe K. Satsuma A. (2016). Ag-M (M: Ni, Co, Cu, Fe) bimetal catalysts prepared by galvanic deposition method for CO oxidation. Catal. Today 265, 2–6. 10.1016/j.cattod.2015.11.043

[B91] Makwana D. Castaño J. Somani R. S. Bajaj H. C. (2020). Characterization of Agar-CMC/Ag-MMT nanocomposite and evaluation of antibacterial and mechanical properties for packaging applications. Arabian J. Chem. 13, 3092–3099. 10.1016/j.arabjc.2018.08.017

[B92] Malik M. A. Albeladi S. S. Al-Maaqar S. M. Alshehri A. A. Al-Thabaiti S. A. Khan I. (2023). Biosynthesis of novel Ag-Cu bimetallic nanoparticles from leaf extract of Salvia officinalis and their antibacterial activity. Life (Basel) 13, 653. 10.3390/life13030653 36983809 PMC10099723

[B93] Malviya K. D. Chattopadhyay K. (2014). Synthesis and mechanism of composition and size dependent morphology selection in nanoparticles of Ag–Cu alloys processed by laser ablation under liquid medium. J. Phys. Chem. C 118, 13228–13237. 10.1021/jp502327c

[B94] Manikam V. R. Cheong K. Y. Razak K. A. (2011). Chemical reduction methods for synthesizing Ag and Al nanoparticles and their respective nanoalloys. Mater. Sci. Eng. B 176, 187–203. 10.1016/j.mseb.2010.11.006

[B95] Manke A. Wang L. Rojanasakul Y. (2013). Mechanisms of nanoparticle-induced oxidative stress and toxicity. BioMed Res. Int. 2013, 1–15. 10.1155/2013/942916 PMC3762079 24027766

[B96] Medina-Cruz D. Saleh B. Vernet-Crua A. Nieto-Argüello A. Lomelí-Marroquín D. Vélez-Escamilla L. Y. (2020). “Bimetallic nanoparticles for biomedical applications: a review,” in Racing for the surface. Editors Li B. Moriarty T. F. Webster T. Xing M. (Cham: Springer International Publishing), 397–434. 10.1007/978-3-030-34471-9_16

[B97] Meghana S. Kabra P. Chakraborty S. Padmavathy N. (2015). Understanding the pathway of antibacterial activity of copper oxide nanoparticles. RSC Adv. 5, 12293–12299. 10.1039/C4RA12163E

[B98] Mei N. Wu Y. Chen B. Zhuang T. Yu X. Sui B. (2022). 3D-printed mesoporous bioactive glass/GelMA biomimetic scaffolds for osteogenic/cementogenic differentiation of periodontal ligament cells. Front. Bioeng. Biotechnol. 10, 950970. 10.3389/fbioe.2022.950970 36329698 PMC9623086

[B99] Metryka O. Wasilkowski D. Mrozik A. (2021). Insight into the antibacterial activity of selected metal nanoparticles and alterations within the antioxidant defence system in *Escherichia coli*, Bacillus cereus and Staphylococcus epidermidis. Int. J. Mol. Sci. 22, 11811. 10.3390/ijms222111811 34769242 PMC8583997

[B100] Modwi A. Taha K. K. Khezami L. Boudina M. Khairy M. Al-Duaij O. K. (2021). Dependence of the electrical properties of Cu-doped ZnO nanoparticles decorated by Ag atoms. Z. Phys. Chemie-Int. J. Res. Phys. Chem. Chem. Phys. 235, 745–767. 10.1515/zpch-2019-1473

[B101] Mohammadi S. Jazani N. H. Kouhkan M. Babaganjeh L. A. (2018). Antibacterial effects of microbial synthesized silver-copper nanoalloys on *Escherichia coli*, Burkholderia cepacia, Listeria monocytogenes and Brucella abortus. Iran. J. Microbiol. 10, 171–179. 30112155 PMC6087701

[B102] Moussa H. Merlin C. Dezanet C. Balan L. Medjahdi G. Ben-Attia M. (2016). Trace amounts of Cu^2+^ ions influence ROS production and cytotoxicity of ZnO quantum dots. J. Hazard Mater 304, 532–542. 10.1016/j.jhazmat.2015.11.013 26619052

[B103] Murugesan G. Latha N. Suganya K. Murugan M. Munusamy M. A. Rajan M. (2018). Stimulus-responsive zinc oxide-functionalized macromolecular humic acid nanocarrier for enhancement of antibacterial activity of ciprofloxacin hydrochloride. Int. J. Biol. Macromol. 114, 1109–1116. 10.1016/j.ijbiomac.2018.03.120 29578024

[B104] Muzikansky A. Nanikashvili P. Grinblat J. Zitoun D. (2013). Ag dewetting in Cu@Ag monodisperse core–shell nanoparticles. J. Phys. Chem. C 117, 3093–3100. 10.1021/jp3109545

[B105] Mvango S. Mashazi P. (2019). Synthesis, characterization of copper oxide-gold nanoalloys and their peroxidase-like activity towards colorimetric detection of hydrogen peroxide and glucose. Mater Sci. Eng. C Mater Biol. Appl. 96, 814–823. 10.1016/j.msec.2018.12.010 30606595

[B106] Nikam A. V. Prasad B. L. V. Kulkarni A. A. (2018). Wet chemical synthesis of metal oxide nanoparticles: a review. CrystEngComm 20, 5091–5107. 10.1039/C8CE00487K

[B107] Noman M. Shahid M. Ahmed T. Niazi M. B. K. Hussain S. Song F. (2020). Use of biogenic copper nanoparticles synthesized from a native Escherichia sp. as photocatalysts for azo dye degradation and treatment of textile effluents. Environ. Pollut. 257, 113514. 10.1016/j.envpol.2019.113514 31706778

[B108] Nyabadza A. Vazquez M. Brabazon D. (2023). A review of bimetallic and monometallic nanoparticle synthesis via laser ablation in liquid. Crystals 13, 253. 10.3390/cryst13020253

[B109] Osowiecki W. T. Ye X. Satish P. Bustillo K. C. Clark E. L. Alivisatos A. P. (2018). Tailoring morphology of Cu-Ag nanocrescents and core-shell nanocrystals guided by a thermodynamic model. J. Am. Chem. Soc. 140, 8569–8577. 10.1021/jacs.8b04558 29909616

[B110] Panchal P. Paul D. R. Sharma A. Choudhary P. Meena P. Nehra S. P. (2020). Biogenic mediated Ag/ZnO nanocomposites for photocatalytic and antibacterial activities towards disinfection of water. J. Colloid Interface Sci. 563, 370–380. 10.1016/j.jcis.2019.12.079 31887701

[B111] Paszkiewicz M. Gołąbiewska A. Rajski Ł. Kowal E. Sajdak A. Zaleska-Medynska A. (2016). Synthesis and characterization of monometallic (Ag, Cu) and bimetallic Ag-Cu particles for antibacterial and antifungal applications. J. Nanomater. 2016, e2187940. 10.1155/2016/2187940

[B112] Pelgrift R. Y. Friedman A. J. (2013). Nanotechnology as a therapeutic tool to combat microbial resistance. Adv. Drug Deliv. Rev. 65, 1803–1815. 10.1016/j.addr.2013.07.011 23892192

[B113] Perdikaki A. Galeou A. Pilatos G. Karatasios I. Kanellopoulos N. K. Prombona A. (2016). Ag and Cu monometallic and Ag/Cu bimetallic nanoparticle-graphene composites with enhanced antibacterial performance. ACS Appl. Mater Interfaces 8, 27498–27510. 10.1021/acsami.6b08403 27680975

[B114] Piao M. J. Kang K. A. Lee I. K. Kim H. S. Kim S. Choi J. Y. (2011). Silver nanoparticles induce oxidative cell damage in human liver cells through inhibition of reduced glutathione and induction of mitochondria-involved apoptosis. Toxicol. Lett. 201, 92–100. 10.1016/j.toxlet.2010.12.010 21182908

[B115] Pourjafari M. Ghane M. Kaboosi H. Sadeghi B. Rezaei A. (2022). Antibacterial properties of Ag–Cu alloy nanoparticles against multidrug-resistant *Pseudomonas aeruginosa* through inhibition of *quorum* sensing pathway and virulence-related genes. J. Biomed. Nanotechnol. 18, 1196–1204. 10.1166/jbn.2022.3331 35854448

[B116] Qing Y. Cheng L. Li R. Liu G. Zhang Y. Tang X. (2018). Potential antibacterial mechanism of silver nanoparticles and the optimization of orthopedic implants by advanced modification technologies. IJN 13, 3311–3327. 10.2147/IJN.S165125 29892194 PMC5993028

[B117] Qiu H. Zhang Z. Huang X. Qu Y. (2011). Dealloying Ag-Al alloy to prepare nanoporous silver as a substrate for surface-enhanced Raman scattering: effects of structural evolution and surface modification. ChemPhysChem 12, 2118–2123. 10.1002/cphc.201100205 21626645

[B118] Qu L.-L. Li Y.-T. Li D.-W. Xue J.-Q. Fossey J. S. Long Y.-T. (2013). Humic acids-based one-step fabrication of SERS substrates for detection of polycyclic aromatic hydrocarbons. Analyst 138, 1523. 10.1039/c2an36764e 23340517

[B119] Radwan-Pragłowska J. Janus Ł. Piątkowski M. Bogdał D. Matysek D. (2020). 3D hierarchical, nanostructured chitosan/PLA/HA scaffolds doped with TiO2/Au/Pt NPs with tunable properties for guided bone tissue engineering. Polymers 12, 792. 10.3390/polym12040792 32252290 PMC7240598

[B120] Roy A. Joshi M. Butola B. S. Malhotra S. (2018). Antimicrobial and toxicological behavior of montmorillonite immobilized metal nanoparticles. Mater. Sci. Eng. C 93, 704–715. 10.1016/j.msec.2018.08.029 30274104

[B121] Sabira S. F. Kasabe A. M. Mane P. C. Chaudhari R. D. Adhyapak P. V. (2020). Selective antifungal and antibacterial activities of Ag-Cu and Cu-Ag core-shell nanostructures synthesized *in-situ* PVA. Nanotechnology 31, 485705. 10.1088/1361-6528/ab9da5 32554903

[B122] Sadiq R. Khan Q. M. Mobeen A. Hashmat A. J. (2015). *In vitro* toxicological assessment of iron oxide, aluminium oxide and copper nanoparticles in prokaryotic and eukaryotic cell types. Drug Chem. Toxicol. 38, 152–161. 10.3109/01480545.2014.919584 24896217

[B123] Saifi M. A. Khan W. Godugu C. (2018). Cytotoxicity of nanomaterials: using nanotoxicology to address the safety concerns of nanoparticles. Pharm. Nanotechnol. 6, 3–16. 10.2174/2211738505666171023152928 29065848

[B124] Satya Bharati M. S. Chandu B. Rao S. V. (2019). Explosives sensing using Ag-Cu alloy nanoparticles synthesized by femtosecond laser ablation and irradiation. RSC Adv. 9, 1517–1525. 10.1039/c8ra08462a 35518042 PMC9059630

[B125] Sharma G. Kumar A. Sharma S. Naushad Mu. Prakash Dwivedi R. Alothman Z. A. (2019). Novel development of nanoparticles to bimetallic nanoparticles and their composites: a review. J. King Saud Univ. - Sci. 31, 257–269. 10.1016/j.jksus.2017.06.012

[B126] Siddiqi K. S. Husen A. (2020). Current status of plant metabolite-based fabrication of copper/copper oxide nanoparticles and their applications: a review. Biomater. Res. 24, 11. 10.1186/s40824-020-00188-1 32514371 PMC7268245

[B127] Sidhu A. Bala A. Singh H. Ahuja R. Kumar A. (2020). Development of MgO-sepoilite nanocomposites against phytopathogenic fungi of rice (oryzae sativa): a green approach. ACS Omega 5, 13557–13565. 10.1021/acsomega.0c00008 32566820 PMC7301367

[B128] Simon T. Bouchonville N. Berr M. J. Vaneski A. Adrović A. Volbers D. (2014). Redox shuttle mechanism enhances photocatalytic H2 generation on Ni-decorated CdS nanorods. Nat. Mater 13, 1013–1018. 10.1038/nmat4049 25087066

[B129] Singhal P. Jha S. K. Pandey S. P. Neogy S. (2017). Rapid extraction of uranium from sea water using Fe 3 O 4 and humic acid coated Fe 3 O 4 nanoparticles. J. Hazard. Mater. 335, 152–161. 10.1016/j.jhazmat.2017.04.043 28448878

[B130] Sun Y. Jiang X. Liu Y. Liu D. Chen C. Lu C. (2021). Recent advances in Cu(II)/Cu(I)-MOFs based nano-platforms for developing new nano-medicines. J. Inorg. Biochem. 225, 111599. 10.1016/j.jinorgbio.2021.111599 34507123

[B131] Tao S. Yang M. Chen H. Ren M. Chen G. (2017). Microfluidic synthesis of Ag@Cu2O core-shell nanoparticles with enhanced photocatalytic activity. J. Colloid Interface Sci. 486, 16–26. 10.1016/j.jcis.2016.09.051 27689722

[B132] Targhi A. A. Moammeri A. Jamshidifar E. Abbaspour K. Sadeghi S. Lamakani L. (2021). Synergistic effect of curcumin-Cu and curcumin-Ag nanoparticle loaded niosome: enhanced antibacterial and anti-biofilm activities. Bioorg. Chem. 115, 105116. 10.1016/j.bioorg.2021.105116 34333420

[B133] Teixeira S. Delerue-Matos C. Santos L. (2019). Application of experimental design methodology to optimize antibiotics removal by walnut shell based activated carbon. Sci. Total Environ. 646, 168–176. 10.1016/j.scitotenv.2018.07.204 30056227

[B134] Tojo C. Vila-Romeu N. (2014). Kinetic study on the formation of bimetallic core-shell nanoparticles via microemulsions. Mater. (Basel) 7, 7513–7532. 10.3390/ma7117513 PMC5510057 28788260

[B135] Torres-Urquidy O. Bright K. (2012). Efficacy of multiple metals against copper-resistant bacterial strains. J. Appl. Microbiol. 112, 695–704. 10.1111/j.1365-2672.2012.05245.x 22277101

[B136] Treguer M. de Cointet C. Remita H. Khatouri J. Mostafavi M. Amblard J. (1998). Dose rate effects on radiolytic synthesis of Gold−Silver bimetallic clusters in solution. J. Phys. Chem. B 102, 4310–4321. 10.1021/jp981467n

[B137] Tsai C.-H. Chen S.-Y. Song J.-M. Chen I.-G. Lee H.-Y. (2013). Thermal stability of Cu@Ag core-shell nanoparticles. Corros. Sci. 74, 123–129. 10.1016/j.corsci.2013.04.032

[B138] Tsuji M. Hikino S. Tanabe R. Matsunaga M. Sano Y. (2010). Syntheses of Ag/Cu alloy and Ag/Cu alloy core Cu shell nanoparticles using a polyol method. CrystEngComm 12, 3900. 10.1039/c0ce00064g

[B139] Tun P. P. Wang J. Khaing T. T. Wu X. Zhang G. (2020). Fabrication of functionalized plasmonic Ag loaded Bi2O3/montmorillonite nanocomposites for efficient photocatalytic removal of antibiotics and organic dyes. J. Alloys Compd. 818, 152836. 10.1016/j.jallcom.2019.152836

[B140] Usman M. S. El Zowalaty M. E. Shameli K. Zainuddin N. Salama M. Ibrahim N. A. (2013). Synthesis, characterization, and antimicrobial properties of copper nanoparticles. Int. J. Nanomedicine 8, 4467–4479. 10.2147/IJN.S50837 24293998 PMC3839804

[B141] Valdez-Salas B. Beltrán-Partida E. Zlatev R. Stoytcheva M. Gonzalez-Mendoza D. Salvador-Carlos J. (2021). Structure-activity relationship of diameter controlled Ag@Cu nanoparticles in broad-spectrum antibacterial mechanism. Mater Sci. Eng. C Mater Biol. Appl. 119, 111501. 10.1016/j.msec.2020.111501 33321601

[B142] Valodkar M. Modi S. Pal A. Thakore S. (2011). Synthesis and anti-bacterial activity of Cu, Ag and Cu–Ag alloy nanoparticles: a green approach. Mater. Res. Bull. 46, 384–389. 10.1016/j.materresbull.2010.12.001

[B143] van Hengel I. a. J. Tierolf M. W. a. M. Valerio V. P. M. Minneboo M. Fluit A. C. Fratila-Apachitei L. E. (2020). Self-defending additively manufactured bone implants bearing silver and copper nanoparticles. J. Mater Chem. B 8, 1589–1602. 10.1039/c9tb02434d 31848564

[B144] Ventola C. L. (2015). The antibiotic resistance crisis: part 1: causes and threats. P T 40, 277–283. 25859123 PMC4378521

[B145] Vincent M. Hartemann P. Engels-Deutsch M. (2016). Antimicrobial applications of copper. Int. J. Hyg. Environ. Health 219, 585–591. 10.1016/j.ijheh.2016.06.003 27318723

[B146] Waiskopf N. Ben-Shahar Y. Banin U. (2018). Photocatalytic hybrid semiconductor-metal nanoparticles; from synergistic properties to emerging applications. Adv. Mater 30, e1706697. 10.1002/adma.201706697 29656489

[B147] Wang X. Li R. Li Z. Xiao R. Chen X.-B. Zhang T. (2019). Design and preparation of nanoporous Ag–Cu alloys by dealloying Mg–(Ag,Cu)–Y metallic glasses for antibacterial applications. J. Mat. Chem. B 7, 4169–4176. 10.1039/C9TB00148D

[B148] Xie Y. Chen S. Peng X. Wang X. Wei Z. Richardson J. J. (2022). Alloyed nanostructures integrated metal-phenolic nanoplatform for synergistic wound disinfection and revascularization. Bioact. Mater 16, 95–106. 10.1016/j.bioactmat.2022.03.004 35386317 PMC8958420

[B149] Xiong Z. Qin F. Huang P.-S. Nettleship I. Lee J.-K. (2016). Effect of synthesis techniques on crystallization and optical properties of Ag-Cu bimetallic nanoparticles. JOM 68, 1163–1168. 10.1007/s11837-015-1757-1

[B150] Yan J. Xia D. Zhou W. Li Y. Xiong P. Li Q. (2020). pH-responsive silk fibroin-based CuO/Ag micro/nano coating endows polyetheretherketone with synergistic antibacterial ability, osteogenesis, and angiogenesis. Acta Biomater. 115, 220–234. 10.1016/j.actbio.2020.07.062 32777292

[B151] Yang L. Chen L. Chen Y.-C. Kang L. Yu J. Wang Y. (2019a). Homogeneously alloyed nanoparticles of immiscible Ag-Cu with ultrahigh antibacterial activity. Colloids Surf. B Biointerfaces 180, 466–472. 10.1016/j.colsurfb.2019.05.018 31100673

[B152] Yang X. Yu Q. Gao W. Tang X. Yi H. Tang X. (2022). The mechanism of metal-based antibacterial materials and the progress of food packaging applications: a review. Ceram. Int. 48, 34148–34168. 10.1016/j.ceramint.2022.08.249 36059853 PMC9419445

[B153] Yang Z. Ma C. Wang W. Zhang M. Hao X. Chen S. (2019b). Fabrication of Cu2O-Ag nanocomposites with enhanced durability and bactericidal activity. J. Colloid Interface Sci. 557, 156–167. 10.1016/j.jcis.2019.09.015 31520996

[B154] Yin I. X. Zhao I. S. Mei M. L. Li Q. Yu O. Y. Chu C. H. (2020). Use of silver nanomaterials for caries prevention: a concise review. IJN 15, 3181–3191. 10.2147/IJN.S253833 32440117 PMC7212989

[B155] Zain N. M. Stapley A. G. F. Shama G. (2014). Green synthesis of silver and copper nanoparticles using ascorbic acid and chitosan for antimicrobial applications. Carbohydr. Polym. 112, 195–202. 10.1016/j.carbpol.2014.05.081 25129735

[B156] Zaleska-Medynska A. Marchelek M. Diak M. Grabowska E. (2016). Noble metal-based bimetallic nanoparticles: the effect of the structure on the optical, catalytic and photocatalytic properties. Adv. Colloid Interface Sci. 229, 80–107. 10.1016/j.cis.2015.12.008 26805520

[B157] Zhang H. Wang C. Li H. Jiang L. Men D. Wang J. (2018a). Physical process-aided fabrication of periodic Au-M (M = Ag, Cu, Ag-Cu) alloyed nanoparticle arrays with tunable localized surface plasmon resonance and diffraction peaks. RSC Adv. 8, 9134–9140. 10.1039/c7ra13567j 35541865 PMC9078608

[B158] Zhang L. Chen J. Yu W. Zhao Q. Liu J. (2018b). Antimicrobial nanocomposites prepared from montmorillonite/Ag ^+^/quaternary ammonium nitrate. J. Nanomater. 2018, 1–7. 10.1155/2018/6190251

[B159] Zhang Z. Zhang Y. (2018). “Introduction of bimetallic nanostructures,” in Bimetallic nanostructures. Editor Zhang Y. (United States: Wiley), 1–22. 10.1002/9781119214618.ch1

[B160] Zhang Z.-Z. Xu J.-J. Shi Z.-J. Cheng Y.-F. Ji Z.-Q. Deng R. (2017). Short-term impacts of Cu, CuO, ZnO and Ag nanoparticles (NPs) on anammox sludge: CuNPs make a difference. Bioresour. Technol. 235, 281–291. 10.1016/j.biortech.2017.03.135 28371766

[B161] Zhao Y. Sarhan R. M. Eljarrat A. Kochovski Z. Koch C. Schmidt B. (2022). Surface-Functionalized Au–Pd nanorods with enhanced photothermal conversion and catalytic performance. ACS Appl. Mat. Interfaces 14, 17259–17272. 10.1021/acsami.2c00221 35389208

[B162] Zhou F. Kostantin E. Yang D.-Q. Sacher E. (2022). Cytotoxicity and antibacterial efficacy of AgCu and AgFe NanoAlloys: a comparative study. Antibiot. (Basel) 11, 1737. 10.3390/antibiotics11121737 PMC9774506 36551394

[B163] Zhu M. Liu X. Tan L. Cui Z. Liang Y. Li Z. (2020a). Photo-responsive chitosan/Ag/MoS2 for rapid bacteria-killing. J. Hazard Mater 383, 121122. 10.1016/j.jhazmat.2019.121122 31518801

[B164] Zhu W. Huang W. Zhou W. Qiu Z. Wang Z. Li H. (2020b). Sustainable and antibacterial sandwich-like Ag-Pulp/CNF composite paper for oil/water separation. Carbohydr. Polym. 245, 116587. 10.1016/j.carbpol.2020.116587 32718655

[B165] Zhu W. Li J. Lei J. Li Y. Chen T. Duan T. (2018). Silver nanoparticles incorporated konjac glucomannan-montmorillonite nacre-like composite films for antibacterial applications. Carbohydr. Polym. 197, 253–259. 10.1016/j.carbpol.2018.06.005 30007611

[B166] Zhu Y. Zhou F. Hu J. Yang L. Yang D.-Q. Sacher E. (2021). A facile route to prepare colorless Ag-Cu nanoparticle dispersions with elevated antibacterial effects. Colloid Surf. a-physicochem. Eng. Asp. 626, 127116. 10.1016/j.colsurfa.2021.127116

[B167] Ziabka M. Matysiak K. Cholewa-Kowalska K. Kyziol A. Krolicka A. Sapierzynski R. (2023). *In vitro* and *in vivo* studies of antibacterial coatings on titanium alloy implants for veterinary application. Int. J. Mol. Sci. 24, 8114. 10.3390/ijms24098114 37175821 PMC10179268

